# Epidemiological and evolutionary consequences of different types of CRISPR-Cas systems

**DOI:** 10.1371/journal.pcbi.1010329

**Published:** 2022-07-26

**Authors:** Hélène Chabas, Viktor Müller, Sebastian Bonhoeffer, Roland R. Regoes

**Affiliations:** 1 Institute for Integrative Biology, ETH Zürich, Zürich, Switzerland; 2 Institute of Biology, Eötvös Loránd University, Budapest, Hungary; Johns Hopkins University, UNITED STATES

## Abstract

Bacteria have adaptive immunity against viruses (phages) in the form of CRISPR-Cas immune systems. Currently, 6 types of CRISPR-Cas systems are known and the molecular study of three of these has revealed important molecular differences. It is unknown if and how these molecular differences change the outcome of phage infection and the evolutionary pressure the CRISPR-Cas systems faces. To determine the importance of these molecular differences, we model a phage outbreak entering a population defending exclusively with a type I/II or a type III CRISPR-Cas system. We show that for type III CRISPR-Cas systems, rapid phage extinction is driven by the probability to acquire at least one resistance spacer. However, for type I/II CRISPR-Cas systems, rapid phage extinction is characterized by an a threshold-like behaviour: any acquisition probability below this threshold leads to phage survival whereas any acquisition probability above it, results in phage extinction. We also show that in the absence of autoimmunity, high acquisition rates evolve. However, when CRISPR-Cas systems are prone to autoimmunity, intermediate levels of acquisition are optimal during a phage outbreak. As we predict an optimal probability of spacer acquisition 2 factors of magnitude above the one that has been measured, we discuss the origin of such a discrepancy. Finally, we show that in a biologically relevant parameter range, a type III CRISPR-Cas system can outcompete a type I/II CRISPR-Cas system with a slightly higher probability of acquisition.

## Author summary

CRISPR-Cas systems are adaptive immune systems that use a complex 3-step molecular mechanism to defend prokaryotes against phages. Viral infections of populations defending themselves with CRISPR-Cas can result in rapid phage extinction or in medium-term phage maintenance. To investigate what controls the fate of the phage population, we use mathematical modeling of type I/II and type III CRISPR-Cas systems, and show that two parameters control the epidemiological short-term outcome: the type of CRISPR-Cas systems and CRISPR-Cas probability of resistance acquisition. Furthermore, the latter impacts host fitness. From this, we derive that 1) for both types, CRISPR-Cas acquisition probability is a key predictor of the efficiency and of the cost of a CRISPR-Cas system, 2) during an outbreak, there is an optimal probability of resistance acquisition balancing the cost of autoimmunity and immune efficiency and 3) type I/II CRISPR-Cas systems are likely to evolve higher acquisition probability than type III.

## Introduction

The pressure viruses exert on their host has resulted in the evolution of various anti-viral immune defences, usually classified as innate or adaptive immunity. Adaptive immunity refers to systems that can acquire new specific targets of immunity during the first encounter with a parasite and memorise this resistance. To be efficient, these systems face a control challenge: they must lessen damage caused by the virus by limiting the spread of an infection and while avoiding dangerous autoimmune reactions [1]. This challenge has resulted in a tight regulation of their reactivity and any misregulation is harmful: lower reactivity results in a predisposition to severe infections whereas higher reactivity results in a propensity for autoimmunity.

The concept of adaptive immunity originates from the study of mammalian immune systems. However, it was later discovered that many others organisms carry some forms of adaptive immunity in addition to their innate immunity [2]. In prokaryotes, innate immunity can be represented by Restriction-Modification systems, which are systems that degrade any DNA with a methylation pattern that differs from the chromosomal one [3]. In addition, prokaryotes can also encode adaptive immunity in the form of CRISPR-Cas systems (Clustered Regularly Interspaced Short Palindromic Repeats—CRISPR ASsociated) [4]. These systems are composed of two loci: a CRISPR locus, that can be seen as a heritable library of small sequences derived from previously encountered viruses and a Cas locus that codes for all the proteins required for the system to work [5]. When a cell is infected by a prokaryotic virus (phage), the invader can be detected by some Cas proteins and a sequence of approximately 30–60 bp of its DNA (the protospacer) is integrated into the chromosomal CRISPR locus (where it is called a spacer): this is the acquisition step. Then, the CRISPR locus is transcribed and the spacer RNA is used as a guide to target the invader DNA and, upon matching, to trigger its degradation (expression and interference steps) [4, 6]. There are six known types of CRISPR-Cas systems, among which 3 (type I, II and III) have been extensively studied. Their study has shown that these different types differ in their Cas genes composition, which sometimes results in differences in their molecular mechanism [5, 7]. One of the key difference between different types of CRISPR-Cas systems concerns the biochemistry of interference which affects the probability of phage escape. In all systems, this step relies on Watson-Crick pairing and viruses can escape a spacer by mutating their targeted protospacer [8–10]. However, there are important differences in the number of point mutation required to escape a given spacer.

For type I and II CRISPR-Cas systems, a single mutation can be all that is needed to escape immunity and because phages are fast evolving parasites, the escape from a spacer is usually fast [8–10]. Interestingly, despite this fast escape from spacers, experimental studies between type I/II CRISPR-Cas systems and virulent phages show that in some cases, type I/II CRISPR-Cas system can lead to the rapid extinction of the phage [11] whereas in others cases, the phage remain in the population on an intermediate time-scale [12, 13]. Recently, it was discovered that phage extinction results from the ability of type I/II CRISPR-Cas systems to generate a diversity of spacers across the bacterial population [11, 12]. This diversity of spacers arises during the acquisition step: phage genomes contain hundreds or thousands of potential targets of the CRISPR-Cas systems—so-called *protospacers*—and the choice among them is stochastic (though potential protospacers vary in their probability of being chosen [12, 14]). As a consequence, each acquisition event leads to the acquisition of a different spacer in each prokaryotic cell, which at the population level, generates a diversity of spacers [10–12, 14, 15]. This diversity acts as an epidemiological shield against the spread of newly evolved escape mutants because when an escape mutant evolves against a given spacer, all hosts carrying another spacer still degrade this mutant thereby preventing its spread [16]. Thus, if the spacer diversity across the population is high enough, phage will be driven to extinction rapidly [11]. The efficiency of type I/II CRISPR-Cas immunity (i.e. its ability to rapidly eradicate the phage population) therefore relies on its ability to generate diversity across the bacterial population and it has been shown both theoretically and experimentally that this generation of diversity derives from the probability per infection that a cell acquires a random spacer [17, 18].

On the other hand, type III interference complex is much less sensitive to escape through point mutations and it has been shown that this escape strategy can be considered as negligible [19, 20]. Because of the lack of type III CRISPR-Cas systems that show natural spacer acquisition under laboratory conditions, very little is known about their evolutionary dynamics with virulent phages. Especially, it is unknown how their insensitivity to phage mutation changes the evolutionary dynamics that has been observed for type I/II CRISPR-Cas systems and if the probability of spacer acquisition impacts the epidemiological outcome in the same way.

In addition, CRISPR-Cas systems are also prone to autoimmunity i.e. to the acquisition of a spacer that targets a chromosomal sequence, which triggers its degradation and this is often thought to be lethal [21–25]. Importantly, the level of autoimmunity of a CRISPR-Cas system is related to its acquisition rates [26, 27]. Therefore, it is likely that CRISPR-Cas systems face a similar control challenge as vertebrate adaptive immune systems: too much acquisition increases autoimmunity, but too little acquisition decreases the efficiency of the immune response [28]. This control challenge is evidenced by three observations. First, spacer acquisition is a rare event: one cell in a million for example for the *Streptococcus thermophilus* most active type II CRISPR-Cas system (CR1 locus) [29]. Second, scientists can easily generate CRISPR-Cas systems with higher levels of acquisition but these are not the forms found naturally [17, 26, 27]. Third, even though the study of CRISPR-Cas regulation is in its early stages, it is already clear that acquisition is tightly regulated [30]. However, how the type of a CRISPR-Cas system affects its control challenge is still unclear.

Previous theoretical studies have tried to model the evolutionary dynamics of CRISPR-Cas systems and a virulent phage (e.g. [31–33, 33–35]). Importantly, several of these studies report that high phage escape rates decrease the efficiency of CRISPR-Cas [31, 32], which suggest that type III and type I/II may face a different control challenges when defending against a virulent phage. In addition, other studies focus on long-term coevolution between phages and type I/II CRISPR-Cas systems (e.g. [15, 34, 35]). They show the importance of viral mutation rates, population-wide spacer diversity and spacer acquisition rate for the emergence of a fluctuating coevolution. Finally, a previous theoretical study attempted to explore the CRISPR-Cas control challenge [36] and looked at how the initial conditions of the bacterial population and CRISPR-Cas autoimmunity impacts the optimal acquisition rate. The study found that autoimmunity decreases the optimal acquisition rate and that the optimal acquisition rate is governed by the interactions of the initial size of the bacteria and phages populations. However, in this work, the authors did not account for the differences in molecular mechanisms between different types of CRISPR-Cas systems. In addition, they made two strong assumptions: 1) the infecting phage can not escape a spacer (much like a type III CRISPR-Cas system) and 2) a single acquisition event is sufficient to assure bacterial survival. These two assumptions are inconsistent with the empirical evidence for type I/II CRISPR-Cas systems [11, 16]. Consequently, it remains unclear how type I/II CRISPR-Cas system respond to their control challenge and if different types of CRISPR-Cas systems show a common dynamics, despite their molecular differences.

Studying how the types of CRISPR-Cas system and its probability of spacer acquisition influence the evolutionary dynamics is experimentally challenging as it would require to finely modify CRISPR-Cas acquisition probability for different types of CRISPR-Cas systems and phage mutation rates without altering other biological parameters. We therefore chose mathematical modeling to explore how the type of immune systems and the probability of spacer acquisition changes the epidemiological outcome and the bacterial fitness. Specifically, we developed a stochastic model of the coupled population dynamics and genetics that simulates the early dynamics of a virulent phage outbreak in a population carrying naive CRISPR-Cas immunity. Our model accounts i) for the ability of type I/II CRISPR-Cas systems to generate spacer diversity by the stochastic acquisition of spacers, ii) the difference in sensitivity of type I/II and type III CRISPR-Cas system for phage escape through mutation and iii) for phage clearance resulting from the infection of cells carrying non-escaped spacers. We focus our work on the initial dynamics of an outbreak, i.e. for a time frame during which bacteria only acquire one spacer and phage escapes only one spacer. We do this for the following reasons. First, it has been noticed that some CRISPR-Cas systems drive phage to extinction rapidly and the reasons for this are unclear. Second, the similarity between type I and type II CRISPR-Cas sytems only holds for the acquisition of one spacer: indeed, for additional acquisition of spacers, these two types of CRISPR-Cas systems use different mechanisms [37, 38]. Third, keeping biologically relevant parameters and allowing simultaneously multiple rounds of spacer acquisition is computationally challenging.

We found that in the absence of autoimmunity for type III CRISPR-Cas systems, increasing CRISPR-Cas acquisition probability is beneficial as it increases the probability of generating at least one spacer that can target a phage. However, for type I/II CRISPR-Cas systems, the survival of the phage population is governed by a threshold-like behavior: below a certain probability of acquisition, the phage always persists, whereas above this critical threshold, it always goes extinct and the transition between these two behaviors is rather steep. This is due to a non-linear relationship between CRISPR-Cas probability of spacer acquisition and the diversity of spacers that is generated. We also show that strains with higher acquisition have higher fitness. In the presence of autoimmunity, epidemiological outcomes are not modified, except for very high acquisitions that impair bacterial survival. However, when strains with different probability of acquisition compete during an outbreak, intermediate acquisition probabilities are selected whereas the lowest probability of acquisition is selected for in absence of phage. We also show that lowering the propensity for autoimmunity (for example by the presence of a self/non-self discrimination mechanism) decreases its cost and therefore selects for higher probability of acquisition. Finally, we show that in a range of biologically relevant parameters, selection can favour a type III CRISPR-Cas system with a lower probability of spacer acquisition over a type I/II CRISPR-Cas system with a higher probability of acquisition. We then discuss the implications of these findings for the evolutionary ecology of CRISPR-Cas and phages.

## Results

**Fig 1:**
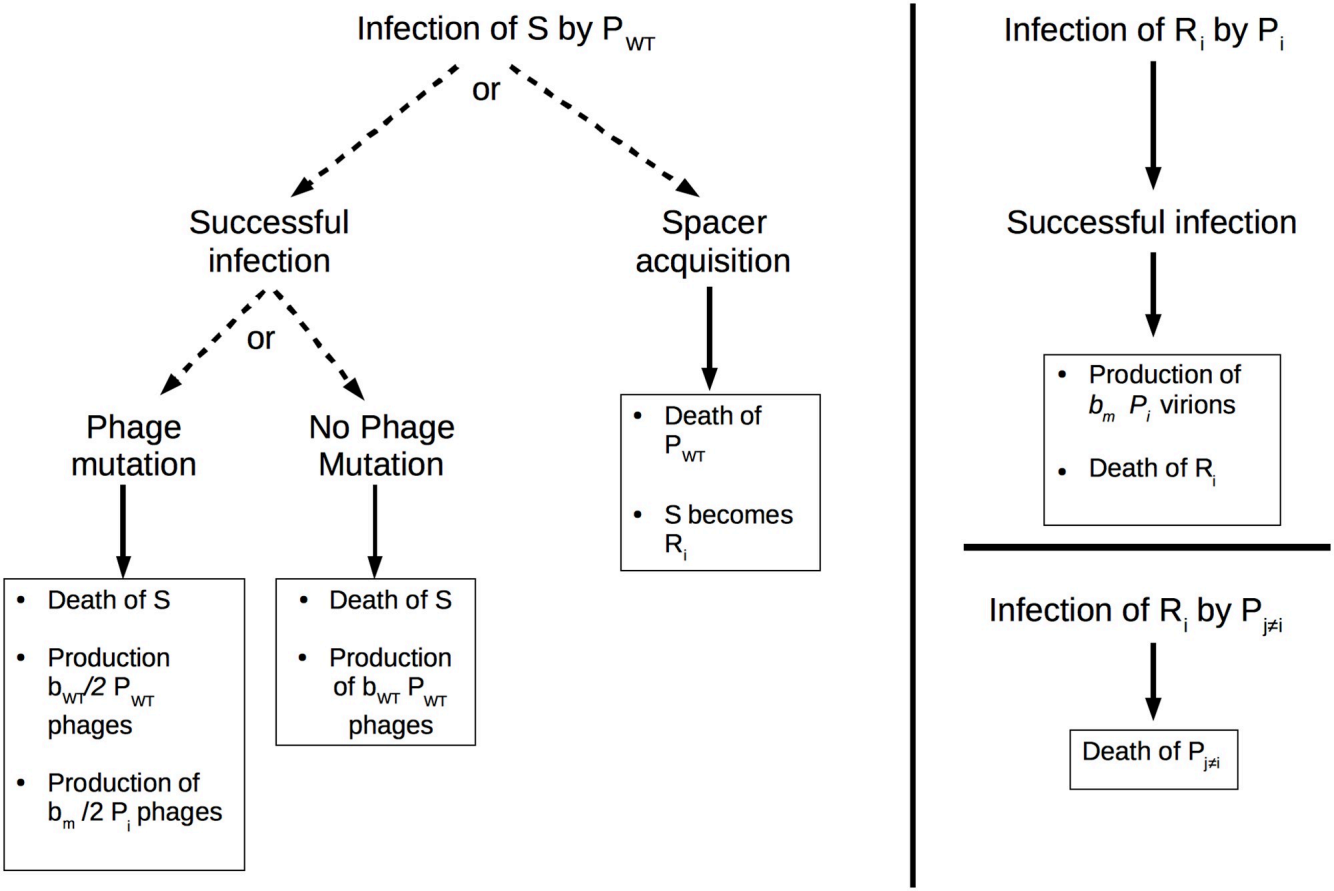
Important transitions in the model.

**Fig 2:**
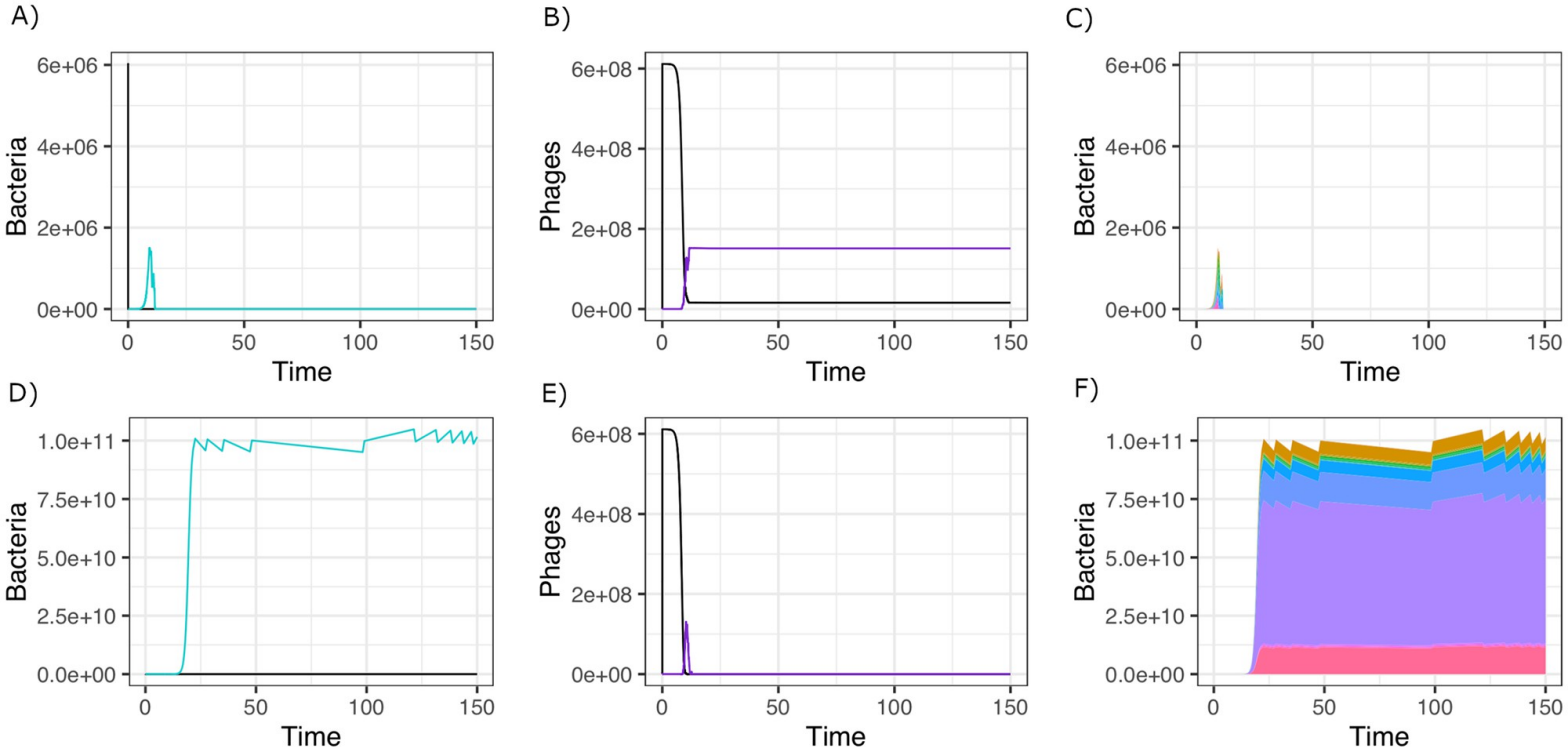
Example of time courses the model produces when 6 * 10^6^ S bacteria are infected by 10^5^ WT phages (*α* = 5.12 * 10^−6^, *μ* = 10^−6^, *b*_*WT*_ = 100, *b*_*m*_ = 70, g = 1.3). Panels A, B and C show the results of a single simulation where the phage drives the bacteria to extinction. Panels D, E and F show the results of a single simulation where the phage is driven to extinction by the CRISPR-Cas system. Panels A and D show the number of bacteria that are sensitive (black) and resistant (blue). Panels B and E show the number of phages that are WT (black) and escape mutants (purple). Panels C and F show the strains diversity of the bacterial population through time, each colour representing a strain.

To study the relationship between CRISPR-Cas acquisition probability, spacer diversity and the outcome of phage infection, we developed a stochastic epidemiological model (see Methods for a full presentation of the model and Fig 1 for a graphic representation). Briefly, the model assumes that naive CRISPR-Cas bacteria *S* are infected by virulent wildtype phage *P*_*WT*_. Infection of naive CRISPR-Cas bacteria leads to one of three outcomes with different probabilities: 1) the phage kills the bacterial cell and produces *P*_*WT*_ progeny, 2) the phage kills the cell and produces *P*_*WT*_ and a randomly-chosen mutated phage *P*_*i*_, 3) CRISPR-Cas kills the infecting phage by acquiring a randomly-selected spacer with an acquisition probability per infection *α*. In addition, *P*_*WT*_ phages can also enter a resistant cell carrying a spacer (cells *R*_*i*_) and this results in phage death, without consequences for the cell. In addition to naive CRISPR-Cas bacteria, escape phages can also infect a cell with a spacer and in this case, the outcome depends on the identity of the spacer and of the escape mutation. If the mutation escapes this specific spacer (*P*_*i*_ infecting *R*_*i*_), the infection kills the cell and produces escape virions, with a fitness cost in the form of a decreased burst size. If the mutation does not escape the spacer the cell carries, *P*_*i*_ infecting *R*_*j*≠*i*_, the phage is killed by the CRISPR-Cas system without consequences for the cell. Escape phages can also infect the naive CRISPR-Cas bacteria, *S*, and this results in their amplification. By changing the probability of phage mutation, the model either describes the interactions between a type I/II CRISPR-Cas system (*μ* = 3.4 * 10^−7^) or a type III CRISPR-Cas system (*μ* = 0). Importantly, when focusing short-term phage survival, neither the bacteria nor the phage evolve in multiple successive rounds: consequently, our model is designed to answer the following question: in which conditions is one round of spacers acquisition sufficient to lead the phage to extinction? These processes have been implemented stochastically and we can therefore follow the dynamics of each genotype, including its appearance and extinction (see Fig 2 for representative simulations).

### The diversity of spacers protects against phage epidemics

To evaluate the validity of our model, we checked whether it confirmed previously observed experimental outcomes. First, we tested whether the model showed that initial spacer diversity protects the bacterial population against phage outbreaks, since it has been shown experimentally that spacer diversity protects the bacterial population by preventing the spread of escape phages [11]. To do so, we ran 100 simulations with an initial bacterial population composed of 50% naive CRISPR-Cas bacteria and 50% resistant cells with various levels of diversity. For this simulation the population was infected by 10^5^ WT phages and we looked at the probability of phage extinction at the end of the simulations. We found (Fig A in S1 Text) that indeed, increasing the initial diversity of spacers in the host population increases the probability of phage extinction in agreement with the experimental results obtained by [11].

### Higher acquisition probabilities lead to higher diversity of spacers

Second, we wanted to confirm that higher probability of acquisition lead to higher levels of spacer diversity. Indeed, Heler et al. found that a mutant with a higher CRISPR-Cas acquisition probability generates more diversity [17]. We ran 100 simulations of an outbreak of an infection with 10^5^ *P*_*WT*_ phages that did not evolve in a naive bacterial population. We looked at the diversity of the bacterial population at the very beginning of the outbreak (at the time when sensitive bacteria *S* would be driven extinct). As expected, we observed that higher acquisition probabilities lead to higher levels of spacers diversity (Fig B in S1 Text, panel A, black line). Notably, we found that the relation between spacer diversity and CRISPR-Cas acquisition probabilities is non-linear. Three regimes can be observed: at low acquisition probabilities, the level of spacer diversity increases slowly; at intermediate probabilities, the diversity increases rapidly and at high probabilities, the system reaches the maximal number of potential spacers and saturates. Therefore, if any increases of spacer diversity are selected for, only increases in spacer acquisition that result in a transition from very low levels to intermediate levels of acquisition or an increase in the intermediate range can lead to an increase in bacterial spacers diversity.

### Phage extinction depends on the type of CRISPR-Cas immunity and on the probability of spacer acquisition

**Fig 3:**
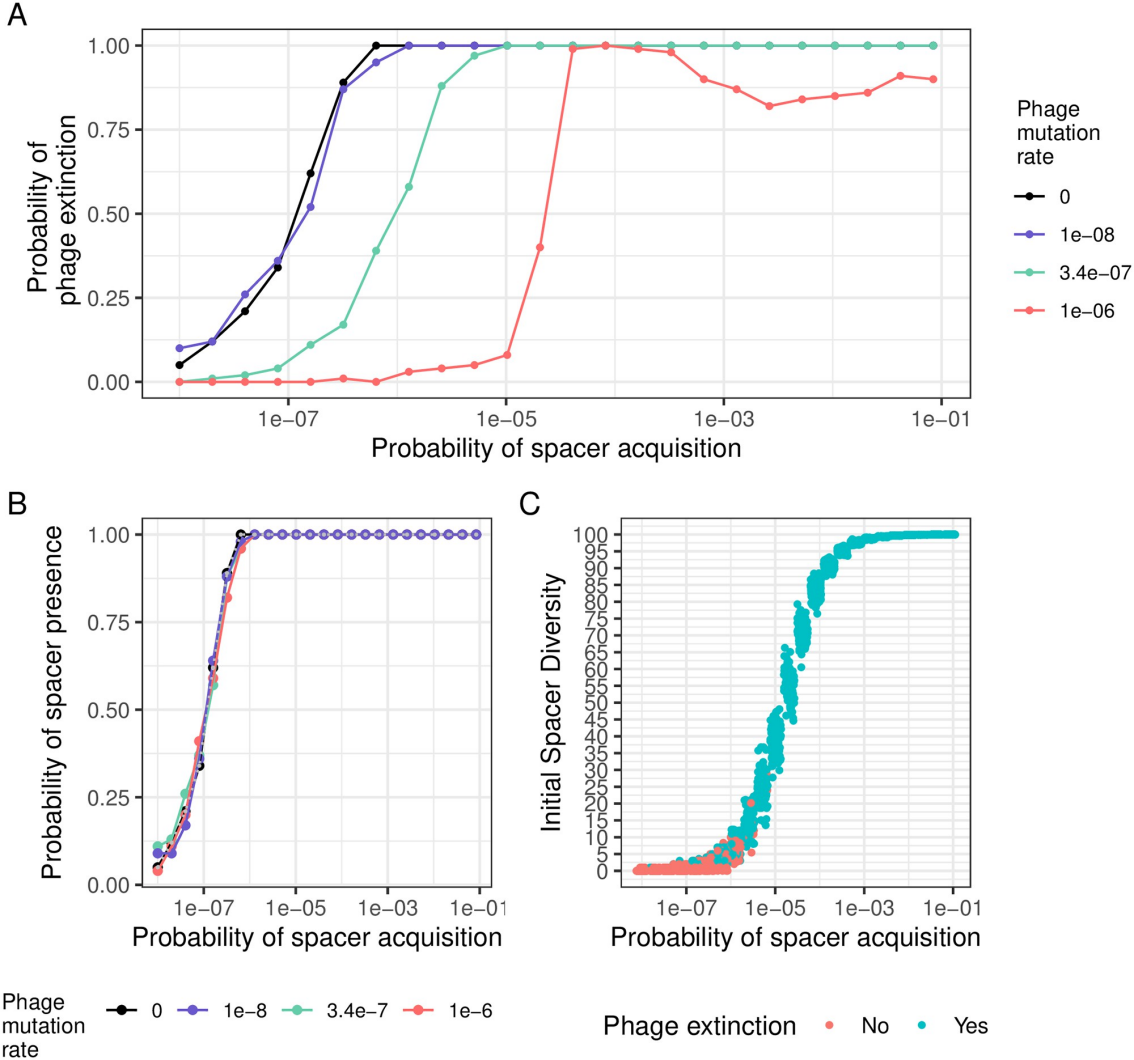
Influence of CRISPR-Cas probability of spacer acquisition on the probability of phage extinction in the absence of autoimmunity. A) Probability of phage survival when infecting bacteria with different acquisition probabilities. The different colours correspond to different levels of phage evolution (*μ*): black, no evolution (Type III CRISPR-Cas systems, *μ* = 0), purple (*μ* = 10^−8^); green (Type I/II CRISPR-Cas systems, *μ* = 3.4 * 10^−7^) and red (*μ* = 10^−6^). B) Probability for bacteria with different probability of spacer acquisition to generate at least one single resistant cell that is present when sensitive cells reach extinction. The different colours correspond to different levels of phage evolution (*μ*): in black, no evolution (Type III CRISPR-Cas systems, *μ* = 0), in purple (*μ* = 10^−8^); in green (Type I/II CRISPR-Cas systems, *μ* = 3.4 * 10^−7^) and in red (*μ* = 10^−6^). The grey line corresponds to the probability of phage extinction in the absence of phage evolution *μ* = 0. C) Relationship between CRISPR-Cas acquisition probability (x-axis), initial spacer diversity (when *S* reaches extinction) (y-axis) and phage extinction (color) for type I/II CRISPR-Cas systems (*μ* = 3.4 * 10^−7^).

To understand the relationship between CRISPR-Cas probability of acquisition and phage extinction, we ran 100 simulations for various acquisition probabilities. For each, we calculated the probability of phage extinction by determining whether at least one of the phage genotype remained at the end of the simulation. We first looked at the outcomes of outbreaks for outbreaks where the bacteria use a type III CRISPR-Cas system to defend (*μ* = 0). We observed that when the probability of spacer acquisition is low, rises in acquisition increase the probability of phage extinction (Fig 3, panel A, black line). When CRISPR-Cas spacer acquisition reaches a certain value (around 10^−6^), the probability of phage extinction saturates to 1. Therefore, up to a certain value, evolving higher probabilities of spacer acquisition enhances the efficacy of type III CRISPR-Cas systems.

However in living systems, phages can also encounter type I/II CRISPR-Cas systems. To see how various types of CRISPR-Cas defense change the epidemiological outcome, we repeated the previous analysis for various probabilities of phage evolution. Indeed, the main difference between type III and type I/II CRISPR-Cas systems, is the possibility for phage to escape spacers by single mutations. As heterogeneity in the escape rates between various spacers targeting the same phage has been previously reported [39], we simulated outbreaks of phages with various escape rates. We found that when phages evolve slowly (*μ* = 10^−8^), the situation does not differ much from the case without evolution (Fig 3, panel A, purple line). However, when spacers escape increases, as it is usually the case with type I/II CRISPR-Cas systems (and *μ* = 3.4 * 10^−7^ and *μ* = 10^−6^), we observed that the outcome of the outbreak was determined by a threshold-like behavior: below a probability of spacer acquisition around 10^−5^ − 5 * 10^−5^, the probability of phage extinction is equal to zero, whereas above this threshold, phages are always driven to extinction by CRISPR-Cas immunity (Fig 3, panel A, red line). It is notable that phage evolution increases the value of the minimal CRISPR-Cas probability of spacer acquisition that ensures phage extinction and sharpen the transition between low and high probabilities of phage extinction: frequent phage evolution puts a high pressure on the CRISPR-Cas system.

While we carefully parameterized our model using all available estimates from the experimental literature, some key parameters have, to our knowledge, not been estimated yet. Most notably, there is no estimate of phage infectivity. To test if different values of infectivity can change these results, we ran the model with various infectivities. Varying the value of the infectivity affected the results (Fig C in S1 Text): a high infectivity increases the minimal probability of spacer acquisition required for phage extinction in all simulations and increase the range of spacer acquisition associated to an intermediate probability of phage extinction.

As in our model, phage mutation results in a progeny containing both WT and escape virions (see Materials and Methods for details), we wondered if this assumption could alter the result of Fig 3. To test for this, we simulated outbreaks with phage mutation resulting in a progeny exclusively composed of escape mutants and we found qualitatively the same results (Fig D in S1 Text). In addition, in natural systems, the cost of a phage mutation can vary [39] but it is assumed to be fixed in our model. We wondered how a change in the cost of escape would alter the epidemiological outcome. To test for this, we repeated the same analysis removing or increasing (mutant burst size equals to 10% of the WT burst size) the phage escape cost. Qualitatively we found the same outcomes except for high phage escape rate where this prevents the CRISPR-Cas system to ensure phage extinction, even at very high probability of spacer acquisition (Fig E in S1 Text).

In addition to purging viruses from the host population, immune systems also prevent the spread of a parasite. To see whether increased CRISPR-Cas probabilities of acquisition decrease the spread of phages, we looked at the size of phage outbreaks for 100 simulations. We found that increasing CRISPR-Cas acquisition probability decreased the size of the outbreak (Fig F in S1 Text). Importantly, increasing CRISPR-Cas acquisition when it is too small to lead the phage to extinction, still resulted in a decrease in the size of the outbreak. As a consequence it seems beneficial for bacteria to evolve higher acquisition, even if these levels do not increase the probability of phage extinction.

### For type III CRISPR-Cas systems, the probability of generating one single spacer drives the epidemiological outcome

How can we explain the previous results? First, we tried to understand how the increase in CRISPR-Cas acquisition probability results in a higher probability of phage extinction for bacteria defending using a type III CRISPR-Cas systems. We reasoned that if the probability of acquisition is too low, there may be a non-zero probability that no bacteria acquire a spacer: in this case the phage would spread in the population until all bacteria have been killed. To check for this, we looked at the number of simulations in which the proportion of resistant strains is positive at the beginning of the outbreak i.e. at the time when the sensitive cells go extinct. We found that indeed increasing the probability of spacer acquisition increased the probability of generating at least one resistant genotype (Fig 3, panel B). Importantly,we found that when phages face a type III CRISPR-Cas system (*μ* = 0), the relationship between the probability of spacer acquisition and the probability of phage extinction is identical to the one between the probability of spacer acquisition and the probability of generating at least one single resistant strain. This means that the probability of generating at least one resistant strain explains the impact of the probability of spacer acquisition on the probability of phage extinction. This also supports that one of the fundamental assumption of the model of [36] is valid for type III CRISPR-Cas systems and therefore suggest that their results may be valid for this specific type of CRISPR-Cas systems.

### For type I/II CRISPR-Cas systems, the probability to generate enough diversity explains the epidemiological outcome

When phage face a type I/II CRISPR-Cas system (*μ* = 3.4*10^−7^), the probability of generating one spacer does not recapitulate the probability of phage extinction. We wondered how the epidemiological critical threshold could be explained. We reasoned that spacer diversity is related to phage extinction and to the probability of spacer acquisition and that it is likely that phages are driven to extinction when initial spacer diversity is high. Because spacer acquisition and phage mutation are stochastic processes, each value of spacer acquisition would then be associated with a probability to generate a certain diversity and each diversity in turn has a certain probability to drive the phages to extinction. Hence we expected that simulations with low initial diversity show a low probability of phage extinction. Indeed, when we looked at the initial spacer diversity depending on the probability of spacer acquisition and at the epidemiological outcome, we observed that simulations with low initial spacer diversity tend to result in phage survival, whereas simulations with high initial spacer diversity tended to result in phage extinction (Fig 3, panel C). Importantly, we observed a diversity critical threshold: above this value, the phage is likely to go extinct whereas below this diversity phage extinction is unlikely.

### In the absence of autoimmunity, evolving higher probabilities of spacer acquisition is always beneficial

**Fig 4:**
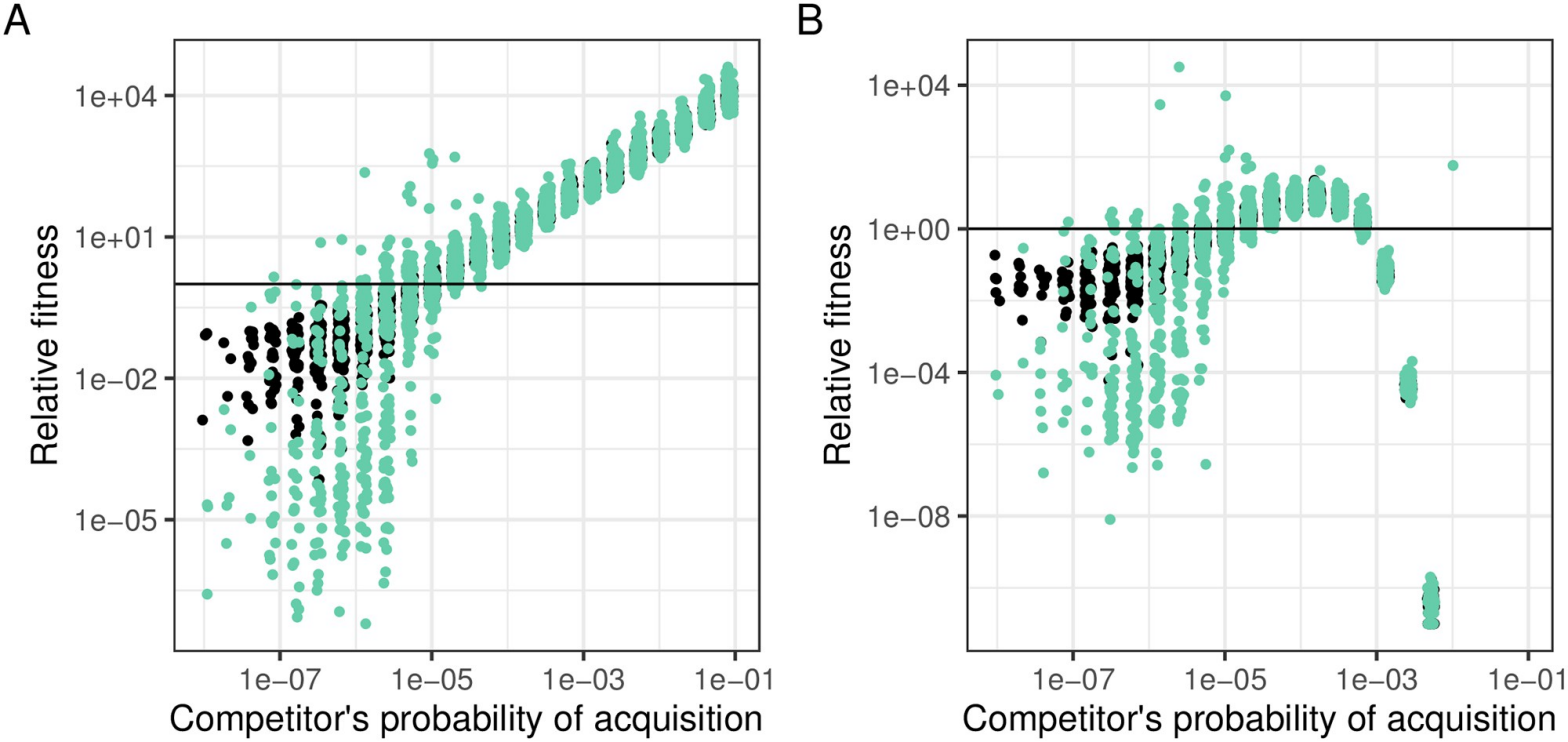
Fitness of a bacterial strain across a range of probabilities of spacer acquisition when in competition with a strain with a probability of spacer acquisition *α* = 10^−5^ in the absence of autoimmunity (A) and in the presence of autoimmunity(B). Colours correspond to the type of CRISPR-Cas immune systems: in black, type III CRISPR-Cas systems (*μ* = 0) and in green, type I/II CRISPR-Cas systems (*μ* = 3.4 * 10^−7^) All simulations were run with Propensity = 40. Each point corresponds to one simulation. Only simulations in which bacteria survive the infection have been plotted.

So far, we looked at the impact of various probabilities of spacer acquisition on the epidemiological outcome. We wondered what the fitness consequences for the bacteria to evolve higher acquisition were. To determine this, we simulated competition experiments with two sensitive strains, that differ only in their probability to acquire a spacer, which were mixed together and were infected with the phage *P*_*WT*_. At the end of the simulation, we calculated the proportion of each strain (sensitive + resistant cells) and we deduced the relative fitness. We observed that it was always beneficial for a strain to evolve higher acquisition probabilities (Fig 4, panel A). However, this is not what is observed in nature, where the probability of acquiring a spacer is usually low [29]. In addition, as one can easily generate CRISPR-Cas systems with higher probabilities of spacer acquisition [17, 26, 27], the absence of strains with high acquisition is likely not the result of evolutionary constraints.

### During an outbreak, autoimmunity selects for intermediate probabilities of spacer acquisition

Natural CRISPR-Cas systems are prone to autoimmunity [21–24], i.e. they can acquire spacers derived from the prokaryote chromosome. This autoimmunity has been shown to be linked to their probability of spacer acquisition and to decrease bacterial fitness [17, 27]. To explore the consequences of this autoimmunity, we added autoimmunity as an additional cause of bacterial death (see Methods for details).

Using this extended model, we looked at the probability of phage extinction for various probabilities of spacer acquisition. We found that the overall outcome of an outbreak is not modified by autoimmunity, except for very high probabilities of acquisition, at which the bacteria are driven to extinction (Fig G in S1 Text). Then we wanted to understand the evolutionary consequences of autoimmunity on bacteria: to do so, we simulated the competition between a WT strain with a probability of spacer acquisition of 10^−5^ and a mutant strain with various acquisition probabilities. At the beginning of the simulation, both strains had equal representation. We observed that in the presence of autoimmunity, intermediate levels of acquisition are selected for (Fig 4, panel B). To get better insights into the cost of autoimmunity, we ran competitions in the absence of phages (Fig H in S1 Text). We observed that it is always beneficial to have a lower probability of spacer acquisition, i.e. that autoimmunity always causes a cost. However, the cost on fitness increases when the probability of spacer acquisition increases: as a consequence, very high acquisitions are strongly selected against, whereas low and intermediate levels of acquisitions are slightly deleterious.

### CRISPR-Cas propensity for autoimmunity determines the optimal probability of acquisition during an outbreak

**Fig 5:**
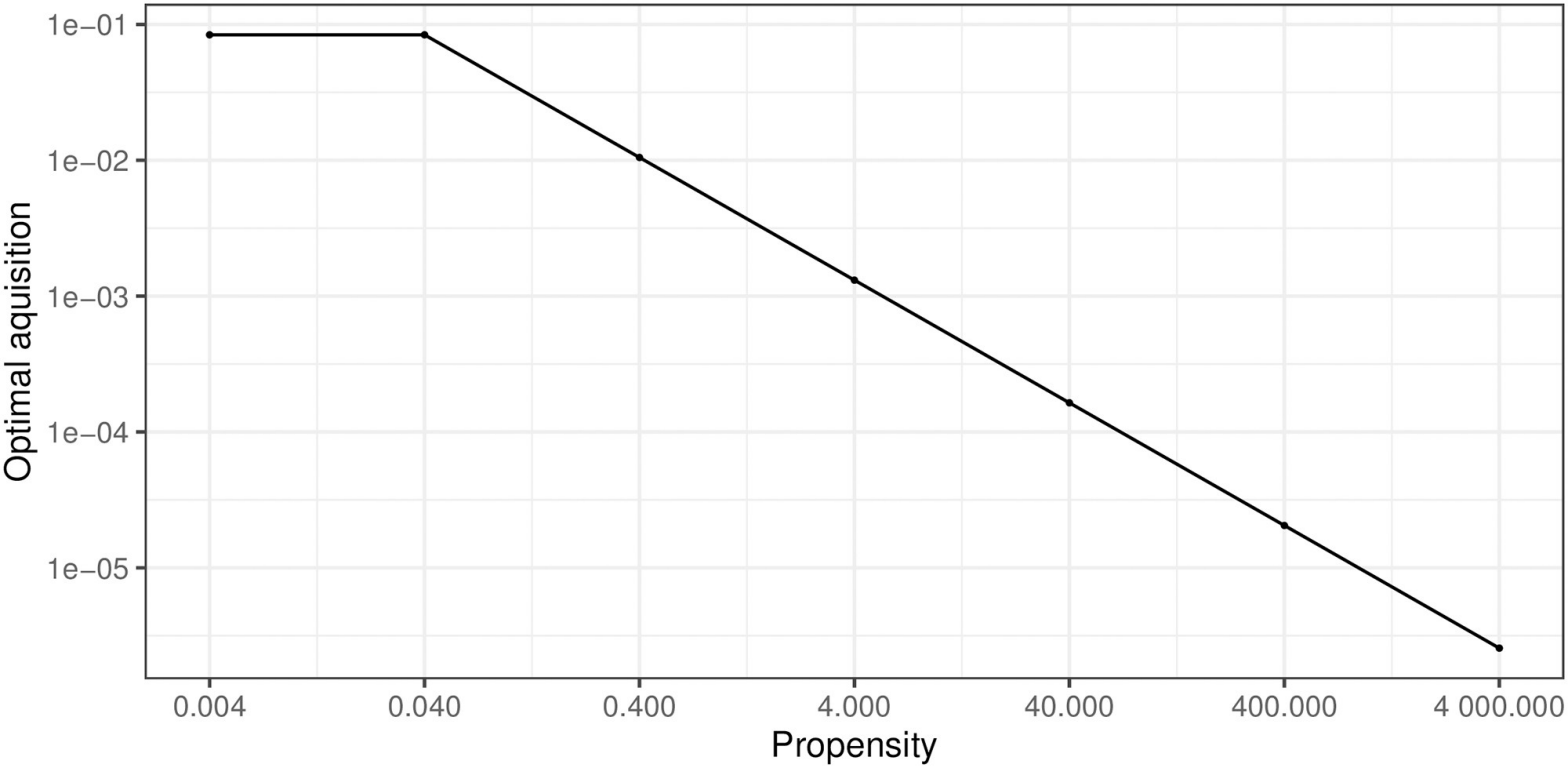
High levels of propensity selects for lower optimal probability of spacer acquisition. A control strain with a probability of acquisition at 10^−5^ competes with a strain of interest, with various acquisition probabilities. Both strains defend using a type I/II CRISPR-Cas system (*μ* = 3.4 * 10^−7^). For each propensity (0.004, 0.04, 0.4, 4, 40, 400 and 4000), we took the median fitness of the bacteria and reported as the optimal probability of spacer acquisition, the probability associated with the highest fitness.

We reasoned that different CRISPR-Cas systems may not have the same propensity for autoimmunity and consequently the cost of autoimmunity can vary independently of the probability of spacer acquisition. To explore the consequences for bacterial fitness of various propensities, we ran 100 competition simulations for various levels of propensity. To the best of our knowledge, there is no quantification of this parameter: we therefore decided to simulate with a propensity ranging from very low (0.004) to very high (4000). We observed that the higher the propensity, the lower the optimal spacer acquisition probability during an outbreak (Fig 5). Interestingly, we observed that high levels of propensity decreases the optimal probability of spacer acquisition at the minimal value needed to assures phage extinction. We wondered then if high levels of propensity could impair the efficiency of CRISPR-Cas immunity. To test for this, we ran 100 simulations of the infection of a bacterial population with high propensity to autoimmunity (*Propensity* = 4000) and look at phage survival. We found that high levels of propensity made really high levels of acquisition unsustainable. However, the effect was not strong enough to prevent phage extinction for intermediate levels of acquisition if phage escape rate is not too high (Fig I in S1 Text).

### Type III CRISPR-Cas systems can outcompete type I/II systems with higher acquisition probabilities

**Fig 6:**
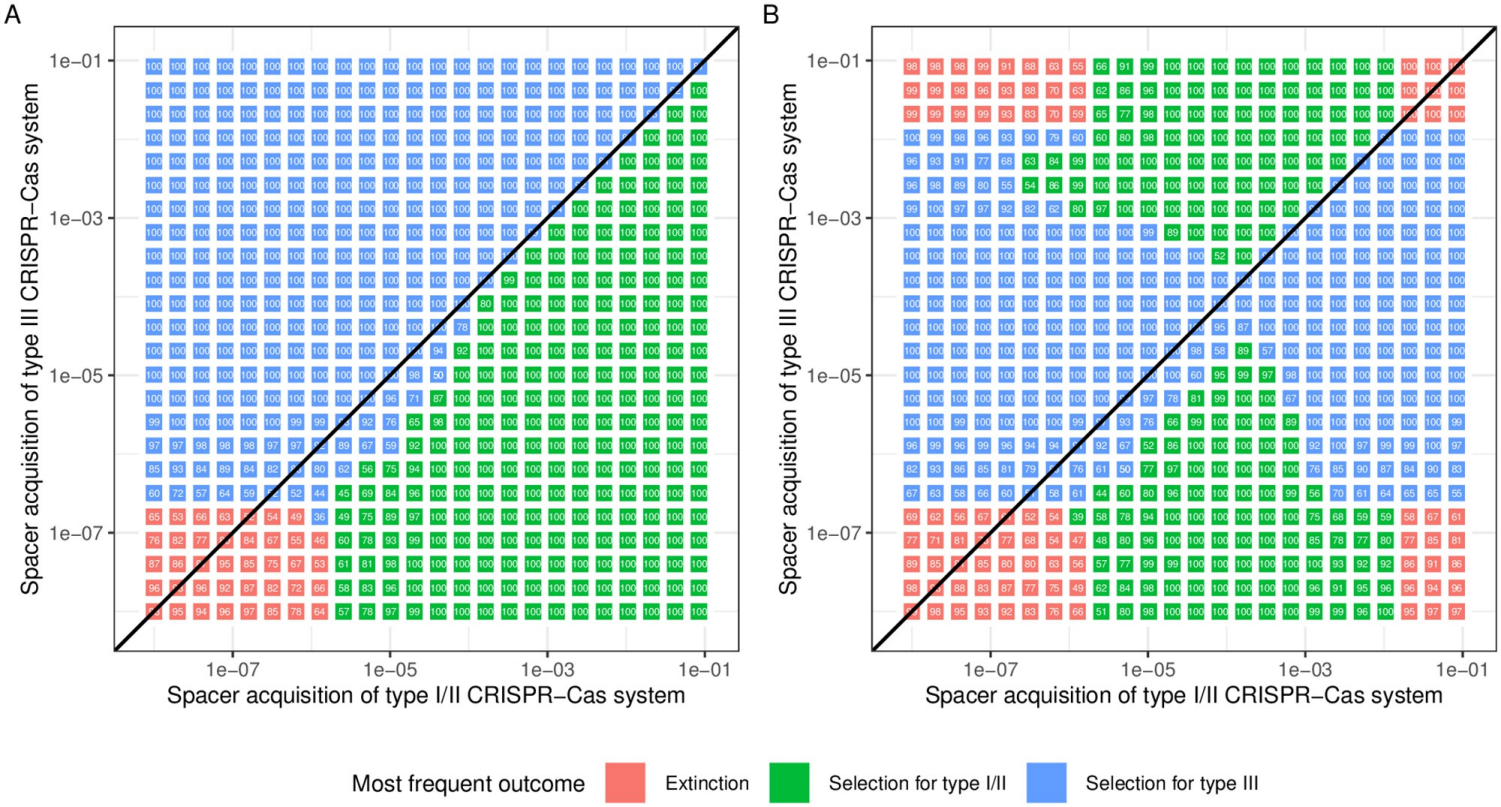
Competitions between type I/II and type III CRISPR-Cas systems. A population of two strains of susceptible bacteria, one carrying a type I/II CRISPR-Cas system and one carrying a type III CRISPR-Cas system, is infected by a virulent phage. For each pair of probability of acquisition, we simulated 100 outbreaks without A) or with B) autoimmunity-induced death (Propensity = 40). We report the most frequent outcome (Extinction of the two strains, Selection for type I/II or Selection for type III) and provide the number of simulations in which this outcome occurred (see numbers in figures).

As we observed a different relationship between the probability of spacer acquisition and the probability of phage extinction depending on the type of CRISPR-Cas system (Fig 3), we wondered if this would result in different competitive ability of the two types of CRISPR-Cas systems. To test for this, we simulated 100 competitions between two strains of bacteria, one carrying a type I/II CRISPR-Cas system and one carrying a type III CRISPR-Cas system. For both strains, we varied the probability of spacer acquisition and we determined the outcome of each simulation (extinction of the two strains, increase of the strain carrying the type I/II system or increase of the strain carrying the type III system). We started by simulating competitions between CRISPR-Cas systems without autoimmunity. In general, we observed that selection favours the system with the higher probability of acquisition (Fig 6A). However, in a biologically relevant range of parameters values (around a probability of acquisition around 10^−6^), we observed that a type III CRISPR-Cas system is selected for, despite a lower probability of spacer acquisition.

To understand how autoimmunity changes these results, we added an autoimmunity-induced death to both CRISPR-Cas systems, as previously described. We observed that autoimmunity changes the outcome of the simulations (Fig 6B): for both systems, there is an optimal probability of acquisition above which the epidemiological benefit is smaller than the cost of autoimmunity and this optimal probability of spacer acquisition is independent of the type of CRISPR-Cas systems. Importantly, we still observed that in a range of biologically relevant parameters (around a probability of acquisition around 10^−6^), type III CRISPR-Cas systems can be selected for despite a lower probability of spacer acquisition.

## Discussion

We studied the control challenge faced by two types of the prokaryotic adaptive immune system, CRISPR-Cas. We found that the outcome of a phage outbreak was governed by CRISPR-Cas probability of spacer acquisition: 1) when resistance escape is impossible, as is the case when phages face type III CRISPR-Cas system, rises in acquisition probability increase the probability of phage extinction and this is governed by the probability for CRISPR-Cas to generate at least one single resistant genotype; 2) when the infected phage has a high rate of resistance escape, as is the case when phages face type I/II CRISPR-Cas systems, phage extinction is controlled by an epidemiological critical threshold: any probability of spacer acquisition below a certain threshold leads to phage persistence whereas any acquisition probability above it leads to phage extinction. Importantly, this critical threshold is above the minimal probability of spacer acquisition that assures phage extinction in the absence of phage evolution. We also found that in the absence of autoimmunity, evolving higher probability of spacer acquisition is always beneficial. However, CRISPR-Cas susceptibility for autoimmunity results in the selection for intermediate acquisition probabilities: indeed, during an outbreak, high levels of acquisition result in high fitness costs that negate the benefits of CRISPR-Cas. Finally, we showed that the optimal probability of spacer acquisition depends on the propensity of the system for autoimmunity.

In this study, we simulated outbreaks of phages facing two types of CRISPR-Cas immune systems, type III and type I/II. This is important because different types of CRISPR-Cas immune systems can have very different biological parameters. For example, even if all types of CRISPR-Cas rely on Watson-Crick pairing to guide the CRISPR complex, their sensitivity to phage mutations varies greatly. For type I and type II systems, a single mutation in the protospacer adjacent motif or in the seed sequence is all that is needed to completely escape the spacer and this usually happens rapidly [8, 16, 39] whereas the interference mechanism of type III CRISPR-Cas systems is resilient to phage mutation and phage escape is extremely rare [19, 20]. Our model reveals important differences in the epidemiological outcomes for type I/II and type III CRISPR-Cas systems. For type I/II CRISPR-Cas systems, the control of phage evolution is mediated by spacer diversity and there is a diversity critical threshold whereas for type III CRISPR-Cas systems, spacer diversity does not impact phage extinction and the epidemiological outcome is driven by the probability to generate at least one resistant genotype. Because the minimal probability of spacer acquisition that ensures to acquire at least one spacer is lower than the one required to reach the epidemiological critical threshold, this would suggest that type III CRISPR-Cas systems can evolve lower probability of spacer acquisition than type I/II CRISPR-Cas systems. Indeed, when we competed type III against type I/II CRISPR-Cas system, we found that in biologically relevant parameter ranges, a type III CRISPR-Cas system with a lower probability of acquisition can outcompete a type I/II system. This result may seem unexpected, as type I CRISPR-Cas systems are much more common in bacteria and archaea than type III CRISPR-Cas systems [40]. We think that these discrepancies between our predictions and the frequency of the various types of CRISPR-Cas systems can have 4 non-mutually exclusive origins. First, carrying type III CRISPR-Cas systems may be costlier for the cell than carrying a type I CRISPR-Cas system in the absence of a phage infection. Second, the frequency of Anti-CRISPR molecules may vary depending on the type of systems. Third, some features of type I CRISPR-Cas systems, such as priming (the boosted acquisition of an additional spacer when the cell carries a partially matching spacer [37, 41]) may also favor type I CRISPR-Cas systems over type III systems. Finally, during interference, type III CRISPR-Cas systems can degrade cell RNAs [42], resulting in a transient growth arrest. This could increase the cost of type III CRISPR-Cas system during a phage outbreak and reverse selection towards type I.

Experimental data to support these model predictions are currently lacking. Even if the importance of spacer diversity for the efficiency of type I/II CRISPR-Cas systems has been documented both experimentally and theoretically [11, 16, 43], the relationship between phage extinction probability and the probability of spacer acquisition has, as far as we know, not been explored. In addition, to the best of our knowledge, there have been no experimental studies of the coevolution of phages and naive type III CRISPR-Cas system. This is most likely due to the lack of type III CRISPR-Cas systems showing naive spacer acquisition under laboratory conditions and this makes the discovery of naive acquisition by the type III CRISPR system of *Thermus thermophilus* very promising [44].

It is striking that, even if we parametrized our model with *S. thermophilus* in mind, our model predicts an epidemiological critical threshold that is around 10^−5^, ten times higher than the probability of spacer acquisition of *S. thermophilus*’ most active CRISPR-Cas system [29]. Importantly, when infected by a virulent phage, *S. thermophilus*, defending exclusively with its CRISPR-Cas systems, survives and leads the phage to extinction in the long term [12, 13] but not in the short term. Our model therefore successfully predicts the epidemiological consequences of such a probability of spacer acquisition. However, our model predicts an optimal probability of spacer acquisition that is two orders of magnitude above *S. thermophilus* CR1 natural acquisition probability. If we assume that the probability of spacer acquisition of this system is as its evolutionary optimum, how can we explain this discrepancy? We think that three non-mutually exclusive hypotheses may explain this. First, a high propensity for autoimmunity could select for an optimal probability of spacer acquisition that is low enough to match biologically relevant values. To the best of our knowledge, there is no estimate of the propensity for chromosomal spacer acquisition versus virulent phages spacer acquisition and it is therefore possible that the values we use are smaller by several order of magnitudes than biological values. Second, the equation modeling the effect of autoimmunity may underestimate its cost. We tried, as best as possible, to model autoimmunity in a way that is biologically relevant (see Methods for details), but experimental data on the relationship between the probability of spacer acquisition and autoimmunity are scarce. Third, as we are interested in the short-term outcome, we are making the assumption that the optimal probability of spacer acquisition is not influenced by longer-term dynamics. However, CRISPR-Cas systems can acquire multiple spacers against a single phage and this may be important for the probability of phage extinction and for the optimal acquisition probability. In addition, many CRISPR-Cas systems display some form of priming, which boosts the acquisition of a second spacer, either because the cell already carries a partially matching spacer (type I) or because it already possesses an efficient spacer (type II) [37, 38, 41]. The impact of these on the relationship between CRISPR-Cas acquisition, the epidemiological outcome, autoimmunity and prokaryote fitness remain to be explored and if these mechanisms are important contributors to the efficiency of the system and of its fitness consequences, it is likely that they change the value of the optimal probability of acquisition. We believe that these are the most parsimonious hypotheses to explain why natural CRISPR-Cas systems have low probability of spacer acquisition while simultaneously, CRISPR-Cas systems with high probability of spacer acquisition can be easily engineered. We therefore call for experimental studies that quantify the propensity for autoimmunity against virulent phages and that study precisely the relationship between the probability of spacer acquisition and chromosomal spacer acquisition and for the theoretical study of the consequences of multiple spacer acquisition (including priming) on the probability of phage extinction and the optimal probability of spacer acquisition.

We also show that in the absence of phages, evolving higher probabilities of acquisition is always costly (Fig H in S1 Text), which selects for CRISPR-Cas systems with the lowest acquisition. As the presence or the absence of phages changes the selection pressure on the optimal acquisition probability, we expect that the optimal probability of spacer acquisition of CRISPR-Cas systems to be lower in environments where phage outbreaks are rare than in environments where they are frequent. One way for CRISPR-Cas systems to respond to this change in selection is to have their acquisition tightly regulated. Studies of the regulation of CRISPR-Cas show that CRISPR-Cas expression is finely regulated [30]. Importantly, during an infection, expression of Cas proteins is increased, which results in higher probability of spacer acquisition. The basal level of acquisition is also dependent on the ecological conditions. For example, it was shown that in some bacteria, CRISPR-Cas acquisition is upregulated by quorum sensing and this has been explained by the higher risk of phage outbreaks when cell density is high [45–47]. This finding makes sense in the light of our predictions as in the absence of phage infection, autoimmunity makes the system always costly and therefore CRISPR-Cas acquisition should be kept at a minimum when the risk of infection is low. Finally, one should not forget that the observed probability of spacer acquisition may also be influenced by abiotic ecological conditions: for example, it has been proposed that infections at lower temperature boosts the probability of spacer acquisition by slowing down the intra-host phage kinetics and therefore letting more time for the system to react [48]. This calls for caution when measuring and studying CRISPR-Cas acquisition as the value measured might be the result of the system itself, its regulation, the infective phage and ecological conditions and may have limited prediction power if any of this changes.

From a phage perspective, we show that lower probability of spacer acquisition increase phage survival both when encoutering type III and type I/II CRISPR-Cas systems. Consequently, it is probably beneficial for phages to decrease CRISPR-Cas acquisition. Many phages carry anti-CRISPR proteins, i.e. small proteins that inhibit some CRISPR-Cas systems [49] anti-CRISPR proteins inhibiting type I, type II and type III CRISPR-Cas systems have been described [50]. To the best of our knowledge, most of them inhibit interference [50], a handful inhibit both acquisition and interference [51] and none inhibit only acquisition: our model suggests that families of anti-CRISPR that decrease CRISPR-Cas acquisition would be beneficial against these three types of CRISPR-Cas systems.

How can we experimentally assess the validity of our model assumptions and predictions? Testing the model predictions and assumptions requires a model system for which the CRISPR-Cas probability of spacer acquisition can be finely tuned. In our view, the most promising system for such work is the *Streptococcus pyogenes* type II CRISPR-Cas system, as its acquisition can be modified by mutations of the Cas9 protein and/or of the long-form tracrRNA [17, 27]. Therefore, mutants with various probabilities of acquisition can be challenged by a virulent phage. As our model only looks at the short term dynamics, such infections should last less than 5 days. At the end of the experiments, phage extinction/survival can be detected by a stamping assay. Given the stochasticity of the outbreak, such an experiment would have to be reproduced sufficiently often to allow the precise quantification of the probability of phage extinction. Such a protocol would assess the existence of the epidemiological critical threshold. In addition, it would also be possible to study the relationship between CRISPR-Cas acquisition, genetic diversity and phage extinction/survival by sequencing the CRISPR array of randomly chosen replicates. Using sequencing data, the spacer diversity can be calculated and the relationship between spacer diversity, spacer acquisition and phage extinction can be assessed. Finally, by knocking-out the nuclease function of Cas9, it is possible to block interference while simultaneously conserving spacer acquisition [52, 53]. Therefore, one can experimentally study the relationship between CRISPR-Cas probability of spacer acquisition and its level of autoimmunity. To do so, mutants with various probabilities of spacer acquisition can have their interference function knocked-out and be grown in the absence of phages. After deep-sequencing of their CRISPR-Cas array, the rate of autoimmunity of each strain can be calculated and variations in autoimmunity levels can be compared to variations in acquisition. As we propose to use a type II CRISPR-Cas system, rapid phage escape is expected. We think that a similar approach using a type III CRISPR-Cas system can be used to assess if the molecular differences between these systems result in the difference in epidemiological outcome that we predict.

Finally, our work focuses on the adaptive immunity of bacteria, but bacteria also carry innate immune systems to defend against phages. One of the most common form of bacterial innate immunity are restriction-modification systems (RM systems). These systems are usually composed of two genes: a methylase that methylate dsDNA and a DNase that degrades any unmethylated dsDNA. Both enzymes target small sequences, called restriction sites, that can be carried by chromosomal or phage DNA. Because the kinetics of these two enzymes is stochastic, autoimmunity, in the form of stochastic degradation of chromosomal DNA, has been reported [54]. They are two key differences with CRISPR-Cas systems regarding DNA interference. First, phages carry several restriction sites on their genome, whereas a given spacer targets only one genetic location on the phage genome. So, even if both CRISPR-Cas and RM systems can be escaped by phage mutation, the complete escape of RM immunity requires the mutation of all the restriction sites the phage carry [55], whereas escaping a type I/II CRISPR-Cas systems require only a single mutation [8]. Second, because the efficiency of RM systems rely on the balance between methylation and restriction, RM systems have a failure rate in the form of the phage DNA being methylated before its degradation. This results in a methylated phage progeny, which can infect all cells carrying a similar RM-system. However, because the phage does not encode the methylase, any infection of a cell not harbouring a similar RM-system results in the loss of this methylation pattern and therefore complex dynamics between RM-system and virulent phages are predicted in bacterial populations carrying a diversity of RM-systems [56].

## Material and methods

### Model definition

In our mathematical model, we consider a bacterial population composed of a sensitive strain *S* carrying a naive CRISPR-Cas system, i. e. with no pre-existing spacers. Upon infection by phage *P*_*WT*_, *S* can acquire a spacer with a probability *α* and evolve resistance against *P*_*WT*_. We assume that the phage genome possesses *n*_*s*_ protospacers and that the CRISPR-Cas system randomly acquires one of them with equal probability. As a result, the infection of *S* will lead to the evolution of a diversified population composed of a subset of resistant strains *R*_*i*_, all carrying a single different spacer (=resistance) against phage WT. All these strains follow a logistic growth with a growth rate *g* and a total carrying capacity *K*.

However, the infection of *S* by *P*_*WT*_ can also be successful because the CRISPR-Cas system fails to stop the infection and the phage reproduces on *S*. A successful infection can either lead to the amplification of *P*_*WT*_ and produce *b*_*WT*_ progeny phages, or result in the escape of the phage from one spacer. Specifically, each protospacer has a probability *μ* to mutate and escape the spacer that targets it. The impact of a mutation on the fraction of the progeny carrying the mutation depends on the phage mode of replication (linear, binary, intermediate). Some phages are known to have a mode of replication that is close to binary whereas others have one close to linear [57] and to the best of our knowledge, the replication mode of the majority of phages is unknown. Therefore, here, we assume an intermediate state where each phage genotype produces half of their respective progeny: $\frac{b_{WT}}{2}$ *P*_*WT*_ and $\frac{b_{m}}{2}$ *P*_*i*_. Each of the escape phage *P*_*i*_ can successfully infect the corresponding resistant bacteria *R*_*i*_ in addition to the sensitive strain *S*. If they infect another resistant strain *R*_*j*_, they are degraded by the CRISPR-Cas system with no consequences for the bacteria. Note that *b*_*m*_ < *b*_*WT*_: this results in escape phage mutants *P*_*i*_ having a lower fitness than *P*_*WT*_, an observation that has been made recently [39]. During amplification of *P*_*WT*_ on S and of *P*_*i*_ on S and *R*_*i*_, we neglect the loss of the infecting phage.

The interaction between bacterial and phage populations described above can be summarized by the following differential equations:

$$\begin{array}{c} \begin{array}{cc} \frac{dS}{dt}= & \underset{\text{Growth}}{\underset{︸}{g_{s}S}}-\underset{\text{Death}}{\underset{︸}{g_{s}S\frac{T}{K}}}-\underset{\text{Predation}\;\text{by}\;\text{WT}\;\text{phages}}{\underset{︸}{(1-\alpha )(1-n_{s}\mu )\beta SP}}-\underset{\text{Predation}\;\text{by}\;\text{escape}\;\text{phages}}{\underset{︸}{\beta S\sum_{j=1}^{n_{s}}P_{i}}}\\ & -\underset{\text{Spacer}\;\text{acquisition}}{\underset{︸}{\alpha \beta SP}}-\underset{\text{Mutation}\;\text{of}\;\text{phage}}{\underset{︸}{(1-\alpha )n_{s}\mu \beta SP}} \end{array} \end{array}$$ (1)

$$\begin{array}{cc} \frac{dR_{i}}{dt}= & \underset{\text{Growth}}{\underset{︸}{g_{r}R_{i}}}-\underset{\text{Death}}{\underset{︸}{g_{r}R_{i}\frac{T}{K}}}+\underset{\text{Spacer}\;\text{acquisition}}{\underset{︸}{\frac{\alpha }{n_{s}}\beta SP}}-\underset{\text{Predation}\;\text{by}\;\text{escape}\;\text{phages}}{\underset{︸}{\beta P_{i}R_{i}}} \end{array}$$ (2)

$$\begin{array}{c} \begin{array}{cc} \frac{dP}{dt}= & \underset{\text{Successful}\;\text{infection}\;\text{of}\;\text{S}}{\underset{︸}{b_{\text{wt}}(1-\alpha )(1-n_{s}\mu )\beta SP}}+\underset{\text{Production}\;\text{of}\;\text{WT}\;\text{when}\;\text{phage}\;\text{escape}}{\underset{︸}{\frac{b_{\text{wt}}}{2}(1-\alpha )n_{s}\mu \beta SP}}\\ & -\underset{\text{Failed}\;\text{infections}\;\text{of}\;\text{R}\;\text{bacteria}}{\underset{︸}{\beta P\sum_{j=1}^{n_{s}}R_{j}}}-\underset{\text{Failed}\;\text{infections}\;\text{when}\;\text{S}\;\text{acquires}\;\text{a}\;\text{spacer}}{\underset{︸}{\alpha \beta SP}} \end{array} \end{array}$$ (3)

$$\begin{array}{cc} \frac{dP_{i}}{dt}= & \underset{\text{Amplification}\;\text{on}\;\text{S}\;\text{and}\;R_{i}\;\text{hosts}}{\underset{︸}{b_{m}\beta P_{i}(S+R_{i})}}+\underset{\text{Mutation}\;\text{of}\;\text{WT}\;\text{Phage}}{\underset{︸}{\frac{b_{m}}{2}\left(1-\alpha \right)\mu \beta SP}}-\underset{\text{Failed}\;\text{infections}\;\text{on}\;\text{resistant}\;\text{bacteria}}{\underset{︸}{\beta P_{i}((\sum_{j=1}^{n_{s}}R_{j})-R_{i})}} \end{array}$$ (4)

We implemented the dynamics described by Eqs 1–4 stochastically. A list of all model variables and parameters and their definition can be found in Table 1. Fig 1 illustrates the different processes we considered in our model.

**Table 1:** Summary of the variables and parameters used in the model.

Variables	Biological interpretation	
*S*	Population size of sensitive bacteria (no spacer)	
*R*_*i*_	Population size of resistant bacteria carrying spacer i	
*T*	Sum of all bacterial population sizes	
*P*	Population size of WT phage (no escape mutation)	
*P*_*i*_	Population size of escape phage (escape mutation in protospacer i)	
Parameters	Biological interpretation	Value
*α*	CRISPR-Cas probability to acquire 1 spacer	variable
*μ*	Mutation probability of a protospacer	variable
*g*	Bacterial growth rate per hour	0.44/*h*
*K*	Bacterial carrying capacity	10^11^
*n*_*s*_	Number of phage protospacers	100
*β*	Infectivity rate constant	10^−6^/*h*
*b*_*WT*_	Burst size of the WT phage (*P*)	190
*b*_*m*_	Burst size of the escape phages (*P*_*i*_)	179
*Propensity*	CRISPR-Cas propensity to autoimmunity	variable

We are aware that our model does not take into consideration some complexities of CRISPR-Cas biology, such as priming [37], acquisition of multiple spacers [4], heterogeneity in the probability of choosing a spacer [14, 58] or multiple phage mutations. In addition, we also did not add a natural phage decay: indeed, each cell can only acquire one spacer, an event that occurs within 24 hours [4, 8, 59]. Consequently, we study outcomes occurring in several days, a timeframe where natural phage decay can be neglected in laboratory conditions.

Overall, our model is similar to the model developed by [15].

### Mathematical modelling of autoimmunity

To test the impact of autoimmunity on bacterial fitness and CRISPR-Cas immunity, we added autoimmunity as an additional cause of bacterial death. To the best of our knowledge, the precise relationship between CRISPR-Cas probability of spacer acquisition and CRISPR-Cas autoimmunity is unknown. However, we know that an increase in acquisition results in higher autoimmunity [26, 27]. In addition, there is limited evidence that an increase in spacer acquisition is proportional to autoimmunity [26]. We therefore modelled the rate of death due to autoimmunity as being proportional to CRISPR-Cas probability of spacer acquisition and to the number of bacteria. In addition, the cost of autoimmunity can be modulated by a parameter representing the propensity of CRISPR-Cas for autoimmunity, independently of its acquisition probability: biologically, this can be understood as the propensity for the bacterial chromosome to be targeted by CRISPR-Cas, for example because of the density of the bacterial chromosome in potential protospacers or in sequences inhibiting acquisition (like Chi sites [26]) or because the bacterial CRISPR-Cas system possesses an efficient self/non-self distinction mechanism.

The rate of death due to autoimmunity is therefore implemented as the following term that is subtracted from Eqs 1 and 2:

$$\begin{array}{c} Autoimmunity=\alpha \times \text{Propensity}\times B\text{,}\;\text{with}\;\text{B}\;\text{being}\;\text{S}\;\text{or}\;R_{i} \end{array}$$

How can we estimate the value of propensity? To the best of our knowledge, two experimental studies have looked at the propensity of CRISPR-Cas systems to acquire spacers from the chromosome or from a resident plasmid and in one study, propensity is estimated to be in the range of 0.001/0.01 whereas in the other one, it is close to 1 [26, 52]. However, it is unclear if these values can be used for phage infection. Indeed, the frequency at which spacers are integrated into the CRISPR locus is also likely to depend on the time the DNA is accessible to the Cas proteins. As phage infections are acute and as spacers are usually integrated at the beginning of the phage life-cycle, phage DNA is accessible for spacer acquisition during a shorter period than chromosomal DNA. To deal with this uncertainty, we chose to use various values for propensity, varying from 0.004 to 4000.

### Choice of parameters values

As we compare our predictions with experimental data, we used as much as possible parameters values that have been measured for a given experimental system comminly use for the study of type II CRISPR-Cas systems: *Streptococcus thermophilus* DGCC7710 and its virulent phage 2972.

Several parameters have been experimentally assessed for the *S. thermophilus*’ system: *μ*, *α* and *b*_*WT*_ [29, 39, 60]. We could also extract values for 3 additional parameters *b*_*m*_, *g* and *n*_*s*_ from experimental data. We determined *b*_*m*_ from the escape phage fitness distribution published in [39]. Briefly, we assumed that all differences in fitness comes from a change in burst size and we derived for each escape phage its burst size (burst time = 40 minutes [61]). We used in our model as *b*_*m*_ the average of the burst size of these escape mutants. Concerning *g*, we derived it from the supplementary figure 2 of [62] (we did not consider the replicate that is different from the others and we used OD2 = 0.5, T2 = 6 hours, OD1 = 0.4 and T2 = 5.5 hours to calculate $r=\frac{ln(\frac{OD2}{OD1})}{T2-T1}$). Finally, *n*_*s*_ corresponds to the potential number of protospacers the phage carries. For type I/II systems, this can be predicted from the PAM sequence of the CRISPR-Cas system. As the CR1 locus of *S. thermophilus* DGCC7710 acquires the majority of spacers against phage 2972 [60], we searched CR1 PAM (AGAA [63]) with SnapGene Viewer 5.3.1 (from Insightful Science; available at snapgene.com) to predict the number of potential protospacers on phage 2972 full genome (Genbank accession number AY699705.1 [64]) and found it to be 413. As using 413 as *n*_*s*_ may be computationally difficult, we chose to decrease this parameter to 100. While choosing this value, we made sure that it was high enough to lead a phage to extinction.

### Bacterial spacer diversity

As a quantitative measure of the bacterial spacer diversity, we calculated the Simpson index from the frequency of each bacterial genotype *p*_*i*_:

$$\begin{array}{c} {}^{2}D=\frac{1}{\sum_{i=1}^{n_{s}}p_{i}^{2}} \end{array}$$ (5)

### Fitness calculation

We calculate relative fitness of two competing bacterial strains from their initial and final frequencies, *p*_*i*_ and *p*_*f*_ using the following formula:

$$\begin{array}{cc} \text{Relative}\;\text{Fitness}= & \frac{p_{f}\left(1-p_{i}\right)}{p_{i}\left(1-p_{f}\right)}\text{,}\;\text{which}\;\text{with}\;p_{i}=0.5 \end{array}$$ (6)

$$\begin{array}{cc} = & \frac{p_{f}}{\left(1-p_{f}\right)} \end{array}$$ (7)

If both strains are going extinct, relative fitness is set to 0. If the control strain goes extinct (i.e. *p*_*f*_ = 1), the relative fitness is equal to “Inf”.

### Model implementation

All simulations and analyses were implemented and conducted in the language for statistical computing R 3.6.3 [65] and the library tidyverse (1.3.0) [66]. To implement the model (Eqs 1–4) stochastically, we used the Gillespie algorithm in the package *adaptivetau* [67].

Except otherwise stated, all simulations were run with the following initial conditions: *S* = 6 * 10^6^ and *P*_*WT*_ = 10^5^, with all others phage and bacterial strains equal to 0. For competition simulations, the initial conditions are: *S*1 = *S*2 = 3 * 10^6^ and *P*_*WT*_ = 10^5^, with all other bacterial and phage strains equal to 0. Parameters values are provided in Table 1. Simulations were terminated when time reaches 150, a time-frame that is long enough for all non-competition simulations to have reached either bacterial or phage extinction.

## Supporting information

S1 Codes In this archive, you will find all the codes used for this study. (ZIP)

S1 Text In this document, we provide supplementary figures. A list of all captions is provided here:
Impact of initial population-wide spacer diversity on the probability of phage extinction.The figure shows the probability of phage extinction in 100 simulations. At the beginning of the simulations, the bacterial population was composed of 6*10^6^ bacteria with an equal representation of sensitive bacteria (S) and different numbers of resistant bacterial genotypes. These populations are infected by 10^5^ P_*WT*_ phages, with phage evolution set to *μ* = 3.4*10^−7^ and the probability of spacer acquisition set to *α* = 0.Influence of CRISPR-Cas probability of spacer acquisition on the mean diversity of newly generated spacers at the beginning (when S goes extinct, panel A) or at the end (panel B) of the outbreak. For simulations resulting in bacterial extinction at the relevant time, we set diversity to 0. We provide the proportion of bacterial extinction at the beginning (panel C) and at the end (panel D) of the simulations.The black curve represents the initial diversity of spacers (when S goes extinct) where phage cannot evolve (*μ* = 0) and the purple, green and red curves when phages can evolve (*μ* = 10^−8^, *μ* = 3.4*10^−7^, *μ* = 10^−6^ respectively). On Panel B, the grey line represents the initial diversity. Error bars correspond to 95% confidence intervals and are barely visible due to limited variation.Influence of phage infectivity on the epidemiological outcome in the absence of autoimmunity.Probability of phage survival when infecting bacteria with various probabilities of spacer acquisition. The different colors correspond to different levels of phage evolution (*μ*): in black, no evolution type III CRISPR-Cas system (*μ* = 0), in purple *μ* = 10^−8^; in green type I/II CRISPR-Cas system *μ* = 3.4*10^−7^ and in red *μ* = 10^−6^. The different panels represent various phage infectivity: A) *β* = 10^−2^, B) *β* = 10^−3^, C) *β* = 10^−4^, D) *β* = 10^−5^, E) *β* = 10^−6^, F) *β* = 10^−7^, G) *β* = 10^−8^.Probability of survival, for a phage infecting bacteria with various probabilities of spacer acquisition when phage mutation results in a progeny exclusively composed of escape mutants.The different colours corresponds to different levels of phage evolution (*μ*): black, type III CRISPR-Cas system (*μ* = 0), purple *μ* = 10^−8^; type I/II CRISPR-Cas system (*μ* = 3.4*10^−7^) and red *μ* = 10^−6^.Influence of the cost of escaping CRISPR-Cas on the probability of phage extinction in the absence of autoimmunity.Probability of phage survival when infecting bacteria with various probabilities of spacer acquisition. The different colours corresponds to different levels of phage evolution (*μ*): black, type III CRISPR-Cas system (*μ* = 0), purple *μ* = 10^−8^; green type I/II CRISPR-Cas system (*μ* = 3.4*10^−7^) and red *μ* = 10^−6^. A) No cost, B) High fitness cost (burst size of mutants equals to 10% of phage WT burst size.)Influence of CRISPR-Cas probability of spacer acquisition on the size of a phage outbreak infecting bacteria using CRISPR-Cas immunity.The colors represent phage evolution: black, type III CRISPR-Cas system (*μ* = 0); purple, green and red *μ* = 10^−8^, type I/II CRISPR-Cas *μ* = 3.4*10^−7^, *μ* = 10^−6^ respectively. Error bars corresponds to 95% confidence intervals.Influence of CRISPR-Cas probability of spacer acquisition on the probability of phage extinction in the presence of autoimmunity.The different colors corresponds to different levels of phage evolution (*μ*): in black, no evolution, type III CRISPR-Cas system (*μ* = 0), in purple *μ* = 10^−8^; in green, type I/II CRISPR-Cas system *μ* = 3.4*10^−7^ and in red *μ* = 10^−6^.Fitness of a bacteria with various probability of spacer acquisition competing against a strain with a probability of spacer acquisition *α* = 10^−5^ in the absence of phages.Each competition has been simulated 100 times and for each of them, the relative fitness has been plotted.Influence of CRISPR-Cas probability of spacer acquisition on the probability of phage extinction with high propensity for autoimmunity (4000).Probability of phage survival when infecting bacteria with various probabilities of spacer acquisition. The different colors corresponds to different levels of phage evolution (*μ*): in black, type III CRISPR-Cas system (*μ* = 0), in purple *μ* = 10^−8^; in green type I/II CRISPR-Cas system *μ* = 3.4*10^−7^ and in red *μ* = 10^−6^.
(PDF)

## References

[1] Bergstrom CT, Antia R. How do adaptive immune systems control pathogens while avoiding autoimmunity? Trends in ecology & evolution. 2006;21(1):22–28. doi: 10.1016/j.tree.2005.11.008 16701466

[2] Müller V, De Boer RJ, Bonhoeffer S, Szathmáry E. An evolutionary perspective on the systems of adaptive immunity. Biological Reviews. 2018;93(1):505–528. doi: 10.1111/brv.12355 28745003

[3] Labrie SJ, Samson JE, Moineau S. Bacteriophage resistance mechanisms. Nature Reviews Microbiology. 2010;8(5):317–327. doi: 10.1038/nrmicro2315 20348932

[4] Barrangou R, Fremaux C, Deveau H, Richards M, Boyaval P, Moineau S, et al. CRISPR provides acquired resistance against viruses in prokaryotes. Science. 2007;315(5819):1709–1712. doi: 10.1126/science.1138140 17379808

[5] Koonin EV, Makarova KS, Zhang F. Diversity, classification and evolution of CRISPR-Cas systems. Current opinion in microbiology. 2017;37:67–78. doi: 10.1016/j.mib.2017.05.008 28605718 PMC5776717

[6] Hampton HG, Watson BN, Fineran PC. The arms race between bacteria and their phage foes. Nature. 2020;577(7790):327–336. doi: 10.1038/s41586-019-1894-8 31942051

[7] Watson BN, Steens JA, Staals RH, Westra ER, van Houte S. Coevolution between bacterial CRISPR-Cas systems and their bacteriophages. Cell Host & Microbe. 2021;29(5):715–725. doi: 10.1016/j.chom.2021.03.018 33984274

[8] Deveau H, Barrangou R, Garneau JE, Labonté J, Fremaux C, Boyaval P, et al. Phage response to CRISPR-encoded resistance in Streptococcus thermophilus. Journal of bacteriology. 2008;190(4):1390–1400. doi: 10.1128/JB.01412-07 18065545 PMC2238228

[9] Semenova E, Jore MM, Datsenko KA, Semenova A, Westra ER, Wanner B, et al. Interference by clustered regularly interspaced short palindromic repeat (CRISPR) RNA is governed by a seed sequence. Proceedings of the National Academy of Sciences. 2011;108(25):10098–10103. doi: 10.1073/pnas.1104144108 21646539 PMC3121866

[10] Sun CL, Barrangou R, Thomas BC, Horvath P, Fremaux C, Banfield JF. Phage mutations in response to CRISPR diversification in a bacterial population. Environmental microbiology. 2013;15(2):463–470. doi: 10.1111/j.1462-2920.2012.02879.x 23057534

[11] van Houte S, Ekroth AK, Broniewski JM, Chabas H, Ashby B, Bondy-Denomy J, et al. The diversity-generating benefits of a prokaryotic adaptive immune system. Nature. 2016;532(7599):385–388. doi: 10.1038/nature17436 27074511 PMC4935084

[12] Paez-Espino D, Morovic W, Sun CL, Thomas BC, Ueda Ki, Stahl B, et al. Strong bias in the bacterial CRISPR elements that confer immunity to phage. Nature communications. 2013;4(1):1–7. doi: 10.1038/ncomms2440 23385575

[13] Common J, Morley D, Westra ER, van Houte S. CRISPR-Cas immunity leads to a coevolutionary arms race between Streptococcus thermophilus and lytic phage. Philosophical Transactions of the Royal Society B. 2019;374(1772):20180098. doi: 10.1098/rstb.2018.0098 30905285 PMC6452269

[14] Heler R, Wright AV, Vucelja M, Doudna JA, Marraffini LA. Spacer acquisition rates determine the immunological diversity of the type II CRISPR-Cas immune response. Cell host & microbe. 2019;25(2):242–249. doi: 10.1016/j.chom.2018.12.016 30709780 PMC6640137

[15] Childs LM, Held NL, Young MJ, Whitaker RJ, Weitz JS. Multiscale model of CRISPR-induced coevolutionary dynamics: diversification at the interface of Lamarck and Darwin. Evolution: International Journal of Organic Evolution. 2012;66(7):2015–2029. doi: 10.1111/j.1558-5646.2012.01595.x 22759281 PMC3437473

[16] Chabas H, Lion S, Nicot A, Meaden S, van Houte S, Moineau S, et al. Evolutionary emergence of infectious diseases in heterogeneous host populations. PLoS biology. 2018;16(9):e2006738. doi: 10.1371/journal.pbio.2006738 30248089 PMC6171948

[17] Heler R, Wright AV, Vucelja M, Bikard D, Doudna JA, Marraffini LA. Mutations in Cas9 enhance the rate of acquisition of viral spacer sequences during the CRISPR-Cas immune response. Molecular cell. 2017;65(1):168–175. doi: 10.1016/j.molcel.2016.11.031 28017588 PMC5218886

[18] Bradde S, Vucelja M, Teşileanu T, Balasubramanian V. Dynamics of adaptive immunity against phage in bacterial populations. PLoS computational biology. 2017;13(4):e1005486. doi: 10.1371/journal.pcbi.1005486 28414716 PMC5411097

[19] Manica A, Zebec Z, Steinkellner J, Schleper C. Unexpectedly broad target recognition of the CRISPR-mediated virus defence system in the archaeon Sulfolobus solfataricus. Nucleic acids research. 2013;41(22):10509–10517. doi: 10.1093/nar/gkt767 24021627 PMC3905844

[20] Pyenson NC, Gayvert K, Varble A, Elemento O, Marraffini LA. Broad targeting specificity during bacterial type III CRISPR-Cas immunity constrains viral escape. Cell host & microbe. 2017;22(3):343–353. doi: 10.1016/j.chom.2017.07.016 28826839 PMC5599366

[21] Stern A, Keren L, Wurtzel O, Amitai G, Sorek R. Self-targeting by CRISPR: gene regulation or autoimmunity? Trends in genetics. 2010;26(8):335–340. doi: 10.1016/j.tig.2010.05.008 20598393 PMC2910793

[22] Jiang W, Bikard D, Cox D, Zhang F, Marraffini LA. RNA-guided editing of bacterial genomes using CRISPR-Cas systems. Nature biotechnology. 2013;31(3):233–239. doi: 10.1038/nbt.2508 23360965 PMC3748948

[23] Vercoe RB, Chang JT, Dy RL, Taylor C, Gristwood T, Clulow JS, et al. Cytotoxic chromosomal targeting by CRISPR/Cas systems can reshape bacterial genomes and expel or remodel pathogenicity islands. PLoS Genet. 2013;9(4):e1003454. doi: 10.1371/journal.pgen.1003454 23637624 PMC3630108

[24] Gomaa AA, Klumpe HE, Luo ML, Selle K, Barrangou R, Beisel CL. Programmable removal of bacterial strains by use of genome-targeting CRISPR-Cas systems. MBio. 2014;5(1). doi: 10.1128/mBio.00928-13 24473129 PMC3903277

[25] Wimmer F, Beisel CL. CRISPR-Cas systems and the paradox of self-targeting spacers. Frontiers in Microbiology. 2020;10:3078. doi: 10.3389/fmicb.2019.03078 32038537 PMC6990116

[26] Levy A, Goren MG, Yosef I, Auster O, Manor M, Amitai G, et al. CRISPR adaptation biases explain preference for acquisition of foreign DNA. Nature. 2015;520(7548):505–510. doi: 10.1038/nature14302 25874675 PMC4561520

[27] Workman RE, Pammi T, Nguyen BT, Graeff LW, Smith E, Sebald SM, et al. A natural single-guide RNA repurposes Cas9 to autoregulate CRISPR-Cas expression. Cell. 2021;184(3):675–688. doi: 10.1016/j.cell.2020.12.017 33421369

[28] Weissman JL, Stoltzfus A, Westra ER, Johnson PL. Avoidance of Self during CRISPR Immunization. Trends in Microbiology. 2020;. doi: 10.1016/j.tim.2020.02.005 32544441

[29] Hynes AP, Lemay ML, Trudel L, Deveau H, Frenette M, Tremblay DM, et al. Detecting natural adaptation of the Streptococcus thermophilus CRISPR-Cas systems in research and classroom settings. nature protocols. 2017;12(3):547–565. doi: 10.1038/nprot.2016.186 28207002

[30] Patterson AG, Yevstigneyeva MS, Fineran PC. Regulation of CRISPR–Cas adaptive immune systems. Current opinion in microbiology. 2017;37:1–7. doi: 10.1016/j.mib.2017.02.004 28359988

[31] Iranzo J, Lobkovsky AE, Wolf YI, Koonin EV. Evolutionary dynamics of the prokaryotic adaptive immunity system CRISPR-Cas in an explicit ecological context. Journal of bacteriology. 2013;195(17):3834–3844. doi: 10.1128/JB.00412-13 23794616 PMC3754601

[32] Weinberger AD, Wolf YI, Lobkovsky AE, Gilmore MS, Koonin EV. Viral diversity threshold for adaptive immunity in prokaryotes. MBio. 2012;3(6):e00456–12. doi: 10.1128/mBio.00456-12 23221803 PMC3517865

[33] Kumar MS, Plotkin JB, Hannenhalli S. Regulated CRISPR modules exploit a dual defense strategy of restriction and abortive infection in a model of prokaryote-phage coevolution. PLoS computational biology. 2015;11(11):e1004603. doi: 10.1371/journal.pcbi.1004603 26544847 PMC4636164

[34] Childs LM, England WE, Young MJ, Weitz JS, Whitaker RJ. CRISPR-induced distributed immunity in microbial populations. PloS one. 2014;9(7):e101710. doi: 10.1371/journal.pone.0101710 25000306 PMC4084950

[35] Pilosof S, Alcala-Corona SA, Wang T, Kim T, Maslov S, Whitaker R, et al. The network structure and eco-evolutionary dynamics of CRISPR-induced immune diversification. Nature Ecology & Evolution. 2020;4(12):1650–1660. doi: 10.1038/s41559-020-01312-z 33077929

[36] Bradde S, Mora T, Walczak AM. Cost and benefits of clustered regularly interspaced short palindromic repeats spacer acquisition. Philosophical Transactions of the Royal Society B. 2019;374(1772):20180095. doi: 10.1098/rstb.2018.0095 30905281 PMC6452266

[37] Fineran PC, Gerritzen MJ, Suárez-Diez M, Künne T, Boekhorst J, van Hijum SA, et al. Degenerate target sites mediate rapid primed CRISPR adaptation. Proceedings of the National Academy of Sciences. 2014;111(16):E1629–E1638. doi: 10.1073/pnas.1400071111 24711427 PMC4000823

[38] Nussenzweig PM, McGinn J, Marraffini LA. Cas9 cleavage of viral genomes primes the acquisition of new immunological memories. Cell host & microbe. 2019;26(4):515–526. doi: 10.1016/j.chom.2019.09.002 31585845 PMC7558852

[39] Chabas H, Nicot A, Meaden S, Westra ER, Tremblay DM, Pradier L, et al. Variability in the durability of CRISPR-Cas immunity. Philosophical Transactions of the Royal Society B. 2019;374(1772):20180097. doi: 10.1098/rstb.2018.0097 30905283 PMC6452261

[40] Makarova KS, Haft DH, Barrangou R, Brouns SJ, Charpentier E, Horvath P, et al. Evolution and classification of the CRISPR–Cas systems. Nature Reviews Microbiology. 2011;9(6):467–477. doi: 10.1038/nrmicro2577 21552286 PMC3380444

[41] Datsenko KA, Pougach K, Tikhonov A, Wanner BL, Severinov K, Semenova E. Molecular memory of prior infections activates the CRISPR/Cas adaptive bacterial immunity system. Nature communications. 2012;3(1):1–7. doi: 10.1038/ncomms1937 22781758

[42] Rostøl JT, Marraffini LA. Non-specific degradation of transcripts promotes plasmid clearance during type III-A CRISPR–Cas immunity. Nature microbiology. 2019;4(4):656–662. doi: 10.1038/s41564-018-0353-x 30692669 PMC6430669

[43] Payne P, Geyrhofer L, Barton NH, Bollback JP. CRISPR-based herd immunity can limit phage epidemics in bacterial populations. Elife. 2018;7:e32035. doi: 10.7554/eLife.32035 29521625 PMC5922976

[44] Artamonova D, Karneyeva K, Medvedeva S, Klimuk E, Kolesnik M, Yasinskaya A, et al. Spacer acquisition by Type III CRISPR–Cas system during bacteriophage infection of Thermus thermophilus. Nucleic Acids Research. 2020;. doi: 10.1093/nar/gkaa685 32821943 PMC7515739

[45] Høyland-Kroghsbo NM, Mærkedahl RB, Svenningsen SL. A quorum-sensing-induced bacteriophage defense mechanism. MBio. 2013;4(1). doi: 10.1128/mBio.00362-12 23422409 PMC3624510

[46] Patterson AG, Jackson SA, Taylor C, Evans GB, Salmond GP, Przybilski R, et al. Quorum sensing controls adaptive immunity through the regulation of multiple CRISPR-Cas systems. Molecular cell. 2016;64(6):1102–1108. doi: 10.1016/j.molcel.2016.11.012 27867010 PMC5179492

[47] Høyland-Kroghsbo NM, Paczkowski J, Mukherjee S, Broniewski J, Westra E, Bondy-Denomy J, et al. Quorum sensing controls the Pseudomonas aeruginosa CRISPR-Cas adaptive immune system. Proceedings of the National Academy of Sciences. 2017;114(1):131–135. doi: 10.1073/pnas.1617415113 27849583 PMC5224376

[48] Høyland-Kroghsbo NM, Muñoz KA, Bassler BL. Temperature, by controlling growth rate, regulates CRISPR-Cas activity in Pseudomonas aeruginosa. MBio. 2018;9(6). doi: 10.1128/mBio.02184-18 30425154 PMC6234860

[49] Borges AL, Davidson AR, Bondy-Denomy J. The discovery, mechanisms, and evolutionary impact of anti-CRISPRs. Annual review of virology. 2017;4:37–59. doi: 10.1146/annurev-virology-101416-041616 28749735 PMC6039114

[50] Wiegand T, Karambelkar S, Bondy-Denomy J, Wiedenheft B. Structures and Strategies of Anti-CRISPR-Mediated Immune Suppression. Annual Review of Microbiology. 2020;74. doi: 10.1146/annurev-micro-020518-120107 32503371 PMC7712631

[51] Vorontsova D, Datsenko KA, Medvedeva S, Bondy-Denomy J, Savitskaya EE, Pougach K, et al. Foreign DNA acquisition by the IF CRISPR–Cas system requires all components of the interference machinery. Nucleic acids research. 2015;43(22):10848–10860. doi: 10.1093/nar/gkv1261 26586803 PMC4678832

[52] Wei Y, Terns RM, Terns MP. Cas9 function and host genome sampling in Type II-A CRISPR–Cas adaptation. Genes & development. 2015;29(4):356–361. doi: 10.1101/gad.257550.114 25691466 PMC4335292

[53] Jinek M, Chylinski K, Fonfara I, Hauer M, Doudna JA, Charpentier E. A programmable dual-RNA–guided DNA endonuclease in adaptive bacterial immunity. science. 2012;337(6096):816–821. doi: 10.1126/science.1225829 22745249 PMC6286148

[54] Pleška M, Qian L, Okura R, Bergmiller T, Wakamoto Y, Kussell E, et al. Bacterial autoimmunity due to a restriction-modification system. Current Biology. 2016;26(3):404–409. doi: 10.1016/j.cub.2015.12.041 26804559

[55] Pleška M, Guet CC. Effects of mutations in phage restriction sites during escape from restriction–modification. Biology letters. 2017;13(12):20170646. doi: 10.1098/rsbl.2017.0646 29237814 PMC5746541

[56] Ruess J, Pleška M, Guet CC, Tkačik G. Molecular noise of innate immunity shapes bacteria-phage ecologies. PLoS computational biology. 2019;15(7):e1007168. doi: 10.1371/journal.pcbi.1007168 31265463 PMC6629147

[57] Sanjuán R, Nebot MR, Chirico N, Mansky LM, Belshaw R. Viral Mutation Rates. Journal of Virology. 2010;84(19):9733–9748. doi: 10.1128/JVI.00694-10 20660197 PMC2937809

[58] Modell JW, Jiang W, Marraffini LA. CRISPR–Cas systems exploit viral DNA injection to establish and maintain adaptive immunity. Nature. 2017;544(7648):101–104. doi: 10.1038/nature21719 28355179 PMC5540373

[59] Cady KC, Bondy-Denomy J, Heussler GE, Davidson AR, O’Toole GA. The CRISPR/Cas adaptive immune system of Pseudomonas aeruginosa mediates resistance to naturally occurring and engineered phages. Journal of bacteriology. 2012;194(21):5728–5738. doi: 10.1128/JB.01184-12 22885297 PMC3486085

[60] Magadán AH, Dupuis MÈ, Villion M, Moineau S. Cleavage of phage DNA by the Streptococcus thermophilus CRISPR3-Cas system. PloS one. 2012;7(7):e40913. doi: 10.1371/journal.pone.0040913 22911717 PMC3401199

[61] Young JC, Dill BD, Pan C, Hettich RL, Banfield JF, Shah M, et al. Phage-induced expression of CRISPR-associated proteins is revealed by shotgun proteomics in Streptococcus thermophilus. PloS one. 2012;7(5):e38077. doi: 10.1371/journal.pone.0038077 22666452 PMC3364186

[62] Vale PF, Lafforgue G, Gatchitch F, Gardan R, Moineau S, Gandon S. Costs of CRISPR-Cas-mediated resistance in Streptococcus thermophilus. Proceedings of the Royal Society B: Biological Sciences. 2015;282(1812):20151270. doi: 10.1098/rspb.2015.1270 26224708 PMC4528535

[63] Horvath P, Romero DA, Coûté-Monvoisin AC, Richards M, Deveau H, Moineau S, et al. Diversity, activity, and evolution of CRISPR loci in Streptococcus thermophilus. Journal of bacteriology. 2008;190(4):1401–1412. doi: 10.1128/JB.01415-07 18065539 PMC2238196

[64] Lévesque C, Duplessis M, Labonté J, Labrie S, Fremaux C, Tremblay D, et al. Genomic organization and molecular analysis of virulent bacteriophage 2972 infecting an exopolysaccharide-producing Streptococcus thermophilus strain. Applied and environmental microbiology. 2005;71(7):4057–4068. doi: 10.1128/AEM.71.7.4057-4068.2005 16000821 PMC1169050

[65] R Core Team. R: A Language and Environment for Statistical Computing; 2018. Available from: https://www.R-project.org/.

[66] Wickham H, Averick M, Bryan J, Chang W, McGowan LD, François R, et al. Welcome to the tidyverse. Journal of Open Source Software. 2019;4(43):1686. doi: 10.21105/joss.01686

[67] Johnson P. adaptivetau: Tau-Leaping Stochastic Simulation; 2019. Available from: https://CRAN.R-project.org/package=adaptivetau.

