# Supplementary material for: Epidemiological and evolutionary consequences of different types of CRISPR-Cas systems
Source: PLoS Comput Biol. 2022 Jul 26;18(7):e1010329. doi: 10.1371/journal.pcbi.1010329 (PMC9355216; doi:10.1371/journal.pcbi.1010329)
Supplement: S1 Codes — (ZIP) [file pcbi.1010329.s001.zip › acquisition-main/Analysis2.html]

Analysis: Epidemiological and evolutionary consequences of different types of CRISPR-Cas systems


Code 

- Show All Code
- Hide All Code

# Analysis: Epidemiological and evolutionary consequences of different types of CRISPR-Cas systems

#### Hélène Chabas, Viktor Müller, Sebastian Bonhoeffer and Roland Regoes

#### January 27, 2022

# 1 Required Packages

These packages need to have been preinstalled before being used.

```
library(tidyverse)
library(ggthemes)
library(patchwork)
```

# 2 Folders

```
# Please add the absolute path of the folder you have stored the data resulting from the simulations. Please note that this folder should only contains the data you want to analyse and nothing else (no other data, no output, no code file).

Data = ""

#Please, add the absolute path of the folder where you want to store the images resulting from the analysis.
Output = ""
```

# 3 Epidemiological outcome

## 3.1 CRISPR Reactivity and probability of phage extinction

This code can be used to make: - Figure 3: panel A (Simulations `No_auto`) - Figure S4 (Simulations `No_auto` with various burstPR) - Figure S6 (Simulations `Auto` with PAM = 40) - Figure S8 (Simulations `Auto` with PAM = 4000) - all panels from Figure S9 (Simulations `No_auto` with various values of beta).

```
Data = ""
setwd(Data)

liste <-list.files(Data,full.names=T) #list all the files in Data

# If you analyse outbreaks without Autoimmunity, please uncomment
data_full = tibble(alpha = c(), mu = c(), burstPR = c(), burstWT = c(), t.extinction = c(), t.extinctionB = c(), Size.epidemics = c(), Nei_i = c(), Nei_f = c())

# If you analyse outbreaks with Autoimmunity, please uncomment
#data_full = tibble(alpha = c(), mu = c(), burstPR = c(), burstWT = c(), t.extinction = c(), t.extinctionB = c(), Size.epidemics = c()) # If you analise Auto

# First, we charge each dataframe from the folder and fuse them in one dataframe.

for (i in levels(factor(liste))){
  data = read_csv(i)
  #########
  #If you analyse outbreaks without autoimmunity, please uncomment:
  data <- tibble(alpha = data$alpha1, mu = data$mu1, burstPR = data$burstPR1, burstWT = data$burstWT1, t.extinction = data$t.extinction, t.extinctionB = data$t.extinctionB, Size.epidemics = data$Size.epidemics, Nei_i = data$Nei_i, Nei_f = data$Nei_f)
  # If you analyse outbreaks with autoimmunity, please uncomment:
  #data <- tibble(alpha = data$alpha1, mu = data$mu1, burstPR = data$burstPR1, burstWT = data$burstWT1, t.extinction = data$t.extinction, t.extinctionB = data$t.extinctionB, Size.epidemics = data$Size.epidemics)
  #########
  data_full = rbind(data_full, data)
}

# Now, we want to modify the data. Indeed, data for which there has been phage extinction have a value different from "Inf" and we want to change this to "Yes". Of course, in case of phage extinction, we want to change this to "No"

data_full <- data_full %>%
             mutate(t.extinction = replace(t.extinction, t.extinction != "Inf", "Yes")) %>%
             mutate(t.extinction =replace(t.extinction, t.extinction == "Inf", "No")) %>%
             mutate(t.extinctionB =replace(t.extinctionB, t.extinctionB != "Inf", "Yes")) %>%
             mutate(t.extinctionB =replace(t.extinctionB, t.extinctionB == "Inf", "No"))

# Now, we want to count the number of simulations for which we had extinction, then calculate the proportion of simulations in which we had extinction and then store this in a new dataframe.

data2 <- data_full %>% 
         group_by(alpha, mu) %>% 
         summarise(extinction = sum(t.extinction == "Yes"), extinctionB = sum(t.extinctionB == "Yes")) %>%
         mutate(Prop = extinction/100, PropB = extinctionB/100)

# And now, we plot the probability of phage extinction and we save the plot in Output

plot_reprot_tot = ggplot(data = data2, aes(x = alpha, y =  Prop, colour = as.factor(mu))) +
       geom_line(size = 0.5) +
       geom_point(size = 1) +
       theme_bw(base_size = 12) +
       theme(legend.position = "right", legend.text = element_text(size=9), legend.title = element_text(size=10)) +
       xlab("Probability of spacer acquisition") +
       ylab("Probability of \n phage extinction") + 
       scale_x_log10() +
       scale_colour_manual(name = " Phage \n mutation \n rate", values = c("black", "slateblue3", "aquamarine3", "indianred1", "blue", "red", "black", "green", "orange"))

#ggsave(plot = plot_reprot_tot, dpi = 300, file = "FigS3_Revisions.pdf", path = Output, width = 19.05, height = 6, units = "cm")
```

## 3.2 CRISPR Reactivity and size of phage outbreaks

This code can be used to generate Fig S5 from data coming from Simulations `No auto`. Please note, that you need to have run the previous section before running this code.

```
data_Size1 <- data_full %>%
              group_by(alpha, mu) %>%
              summarise(size = mean(Size.epidemics), SD = sd(Size.epidemics))

plot.size = ggplot(data = data_Size1, aes(x = alpha, y = size, colour = as.factor(mu))) +
            geom_line(size = 0.5) +
            geom_point(size = 1) +
            geom_errorbar(aes(x = alpha, ymin = size - 1.96*SD/sqrt(100), ymax = size + 1.96*SD/sqrt(100)),width = 0.1) +
            theme_bw(base_size = 12) +
            theme(legend.position = "right") +
            xlab("Probability of spacer acquisition") +
            ylab("Size of phage epidemics") + 
            scale_x_log10() +
      scale_y_log10() +
            scale_colour_manual(name = " Phage \n mutation \n rate", values = c("black", "slateblue3", "aquamarine3", "indianred1", "blue", "red", "black", "green", "orange"))

ggsave(plot.size, filename = "FigS5_Revision.pdf", path = Output, width = 10, height = 6, units = "cm")
```

## 3.3 CRISPR Reactivity and Nei Diversity

This code can be used to make Fig S2 (all panels). Please note that for this, you need data simulated with the code `Simuls No auto` from Simulations.rmd and having run the first section of the present code.

```
# Calculations of the average Nei Diversity for each CRISPR Reactivity and for each phage protospacer mutation

# We calculate the mean Nei Diversity. Because the phage can drive the bacteria to extinction, it is possible that some Nei Diveristy equal to NA which would result in Mean and Variances equal to NA. Therefore, we ignore simulations where bacteria go extinct and we calculate these statistics only for simulations for which Nei is different from NA. 

# If Mean and Var are equals to NA, it means that all simulations resulted in bacterial extinction. We want to replace this NA by the value 0, in order to plot it.


data_Nei = data_full

Nei_Mean <- data_Nei %>%
            group_by(alpha, mu) %>%
            summarise(NeiR1_Mean = mean(Nei_i, na.rm = TRUE), NeiRf_Mean = mean(Nei_f, na.rm = TRUE), repi = 100 - sum(is.na(Nei_i)), repf = 100 - sum(is.na(Nei_f)), NeiR1_Var = 1.96 * sd(Nei_i, na.rm = TRUE) / sqrt(repi), NeiRf_Var = 1.96 * sd(Nei_f, na.rm = TRUE)/sqrt(repf), Exti = sum(is.na(Nei_i == TRUE))/100, Extf = sum(is.na(Nei_f == TRUE))/100) %>%
            mutate(NeiR1_Mean = replace(NeiR1_Mean, is.na(NeiR1_Mean == TRUE), 0), NeiRf_Mean = replace(NeiRf_Mean, is.na(NeiRf_Mean == TRUE), 0), NeiR1_Var = replace(NeiR1_Var, is.na(NeiR1_Var == TRUE), 0), NeiRf_Var = replace(NeiRf_Var, is.na(NeiRf_Var == TRUE), 0))

# Plot probability of bacterial extinction

plotBi = ggplot(data = Nei_Mean, aes(x = alpha, y =  Exti, colour = as.factor(mu))) +
    geom_line(size = 0.5) +
    geom_point(size = 1) +
    theme_bw(base_size = 12) +
    theme(legend.position = "none") +
    xlab("Probability of spacer acquisition") +
    ylab("Probability of bacterial extinction") + 
    scale_x_log10() +
    scale_colour_manual(name = " Phage \n mutation \n rate", values = c("black", "slateblue3", "aquamarine3", "indianred1", "blue", "red", "black", "green", "orange"))

plotBf = ggplot(data = Nei_Mean, aes(x = alpha, y =  Extf, colour = as.factor(mu))) +
    geom_line(size = 0.5) +
    geom_point(size = 1) +
    theme_bw(base_size = 12) +
    theme(legend.position = "none") +
    xlab("Probability of spacer acquisition") +
    ylab("Probability of bacterial extinction") + 
    scale_x_log10() +
    scale_colour_manual(name = " Phage \n mutation \n rate", values = c("black", "slateblue3", "aquamarine3", "indianred1", "blue", "red", "black", "green", "orange"))

# Plot of initial Nei diversity
plot_Nei1 = ggplot(data = Nei_Mean, aes(x = alpha, y = NeiR1_Mean, colour = as.factor(mu))) + 
            geom_point(size = 0.7) +
            geom_line() +
            geom_errorbar(data = Nei_Mean, aes(x = alpha, ymin = NeiR1_Mean-NeiR1_Var, ymax = NeiR1_Mean+NeiR1_Var), width = 0.1) +
            theme_bw(base_size = 12) +
            scale_x_log10() +
            theme(legend.position ="none") +
            ylab("Diversity of bacteria") +
            xlab("Probability of spacer acquisition") +
            scale_colour_manual(name = " Phage \n mutation \n rate", values = c("black", "slateblue3", "aquamarine3", "indianred1", "blue", "red", "black", "green", "orange"))

# Plot final Nei Diversity.
plot_Neif = ggplot() + 
            geom_point(data = Nei_Mean, aes(x = alpha, y = NeiRf_Mean, colour = as.factor(mu)), size = 0.7) +
            geom_point(data = Nei_Mean, aes(x = alpha, y = NeiR1_Mean, colour = as.factor(mu)), colour = "grey", size = 0.7) +
            geom_line(data = Nei_Mean, aes(x = alpha, y = NeiRf_Mean, colour = as.factor(mu))) +
            geom_line(data = Nei_Mean, aes(x = alpha, y = NeiR1_Mean, colour = as.factor(mu)), colour = "grey") +
            geom_errorbar(data = Nei_Mean, aes(x = alpha, ymin = NeiRf_Mean-NeiRf_Var, ymax = NeiRf_Mean+NeiRf_Var, colour = as.factor(mu)), width = 0.1) +
            geom_errorbar(data = Nei_Mean, aes(x = alpha, ymin = NeiR1_Mean-NeiR1_Var, ymax = NeiR1_Mean+NeiR1_Var), width = 0.1, colour = "grey") +
            theme_bw(base_size = 12) +
            scale_x_log10() +
            theme(legend.position ="none") +
            ylab("Diversity of bacteria") +
            xlab("Probability of spacer acquisition") +
            scale_colour_manual(name = " Phage \n mutation \n rate", values = c("black", "slateblue3", "aquamarine3", "indianred1", "grey"))

figS2 = (plot_Nei1 + plot_Neif) / (plotBi + plotBf) + plot_annotation(tag_levels = 'A')

ggsave(plot = figS2, filename = "FigureS2Revision.pdf" , path = Output, width = 19.05, height = 19, units = "cm")
```

## 3.4 CRISPR Reactivity and probability of generating at least one single resistant cell

This code can be used to generate Figure 3, panel B.

You need data coming from Simulations `No_auto`.To run this section, you need to have run the previous section.

```
# As Nei_i corresponds to the Nei diversity when S are extinct, it means that if Nei_i exists, i.e. here Nei_i > 0, one resistant cell at least has been generated. 

data_proba_BIM <- data_full %>%
                  group_by(alpha, mu) %>%
                  summarise(proba_BIM = sum(Nei_i > 0, na.rm = TRUE)/100)

data3 = data2[data2$mu == 0,]

plot_proba = ggplot(data = data_proba_BIM, aes(x = alpha, y = proba_BIM, colour = as.factor(mu))) +
            geom_point()+
            geom_line() +
            geom_point(data = data3, aes(x = alpha, y =  Prop, colour = "grey"), size = 0.2) +
            geom_line(data = data3, aes(x = alpha, y =  Prop, colour = "grey"), linetype = "dotted") +
            theme_bw(base_size = 12) +
            theme(legend.position = "bottom", legend.text = element_text(size=9), legend.title = element_text(size=10)) +
            scale_x_log10() +
            ylab("Probability of spacer presence") +
            xlab("Probability of spacer acquisition") +
            scale_colour_manual(name = " Phage \n mutation \n rate", values = c("black", "slateblue3", "aquamarine3", "indianred1", "grey"), labels = c("0","1e-8","3.4e-7","1e-6", "Extinction"))
```

## 3.5 CRISPR Reactivity, Initial Nei diversity and phage extinction.

This code can be used to make Figure 3, panel C. You need data generated from simulations `No_auto` stored in folder `Data`. The folder must only contain the data you want to analyse. You need to have run the first section of this code before runing this section.

```
data_full_6 = data_full[data_full$mu == 3.4e-7,]

data_full_6 <- data_full_6 %>%
               mutate(Nei_i = replace(Nei_i, is.na(Nei_i == TRUE), 0))

plot.div = ggplot(data = data_full_6, aes(x = alpha, y = Nei_i, group = alpha, colour = t.extinction)) + 
           geom_point(size = 1, position = "jitter") +
           scale_y_continuous(breaks = seq(0, 100, 5), name = "Initial Spacer Diversity") +
           xlab("Probability of spacer acquisition") +
           theme_bw(base_size = 12) +
           theme(legend.position = "bottom") +
           scale_x_log10()+
           scale_colour_discrete(name = "Phage extinction")
```

## 3.6 Initial bacterial diversity and phage extinction

This code is required to make Figure S1. You need data generated with `Diversity` from Simulations.rmd.

```
# Absolute path of folder where the data are stored. Caution, this folder must only contains the data you want to analyse.

Data <- ""

# Absolute path of folder where you want to store figures.
Output <- ""

setwd(Data)

liste <-list.files(Data,full.names=T) #list all files in Data

data_full = tibble(alpha =c(), mu = c(), burstPR = c(), burstWT = c(), div = c(), t.extinction = c(), Size.epidemics = c()) # Required empty dataframe

# Here, for each simulation, we charge the data, determine wheter there has been phage extinction and store this in data_full.
for (i in levels(factor(liste))){
    data <- read_csv(i)
    data <- tibble(alpha = data$alpha1, mu = data$mu1, burstPR = data$burstPR1, burstWT = data$burstWT1, div = data$div, t.extinction = data$t.extinction, Size.epidemics = data$Size.epidemics)
    data_full <- rbind(data_full, data)
}

data_full <- data_full %>%
            mutate(t.extinction = replace(t.extinction, t.extinction != "Inf", "Yes"), t.extinction = replace(t.extinction, t.extinction == "Inf", "No"))

data_div <- data_full %>%
            group_by(alpha, div) %>%
            summarise(Prop = sum(t.extinction == "Yes")/100)

plot = ggplot(data = data_div, aes(x = div, y =  Prop)) +
       geom_line(size = 0.2) +
       geom_point(size = 1) +
       theme_bw(base_size = 7) +
       theme(legend.position = "right") +
       xlab("Diversity of resistant bacteria") +
       ylab("Probability of phage extinction")

ggsave(plot = plot, dpi = 300, file = "FigS1_Revisions.pdf", path = Output, width = 10, height = 6, units = "cm")
```

# 4 Analysis Competition Simulations

This code can be used to make Figure 4 panel A and B and Figure S7.

You require Competition simulations either in absence of autoimmunity `Compet No_auto` (Figure 4, panel A) or in presence of autoimmunity `Compet auto` (Figure 4, panel B) or in presence of autoimmunity but in absence of phage `Compet no Phage` (Figure S7).

```
# Absolute file of the folder containing the data. This folder must only contain the data.
Data_no_auto  = ""

Data_auto = ""

Data_nophage = ""

Data = Data_no_auto # Set Data to Data_no_auto or Data_auto or Data_nophage

setwd(Data)

# Absolute file of the folder where you want to store the graph.
Output = ""

liste <-list.files(Data,full.names=T) # make a list of all files in Data.

data_full = tibble(alpha1 =c(), alpha2 = c(), mu = c(), Prop150_1 = c(), Prop150_2 = c()) # required dataset for analysis.

# Here, we charge the data and save everything in a single dataset.
for (j in levels(factor(liste))){
  data = read_csv(j)
  data <- tibble(alpha1 = data$alpha1, alpha2 = data$alpha2, mu = data$mu, Prop150_1 = data$Prop150_1, Prop150_2 = data$Prop150_2)
  data_full = rbind(data_full, data)
}

data_full <- data_full %>%
              mutate(Fitness = Prop150_2 / (1 - Prop150_2)) %>%
              mutate(Fitness = replace(Fitness, is.na(Fitness) == TRUE, 0))

# If you want to draw Figure 4 panel A or B (i.e. simulations with phages)
plot_fitness_no_auto = ggplot(data = data_full, aes(x = as.numeric(alpha2), y = Fitness, group = interaction(as.factor(alpha2), as.factor(mu)), fill = as.factor(mu), colour = as.factor(mu))) +
               geom_point(position = position_jitter(width = 0.05), size = 1) +
               theme_bw(base_size = 12) +
               theme(legend.position = "none") +
               xlab("Competitor's probability of acquisition") +
               scale_x_log10() +
               scale_y_log10() +
               geom_hline(yintercept = 1, size = 0.4) +
               ylab("Relative fitness") +
               scale_fill_manual(values = c("black", "indianred1")) +
               scale_colour_manual(values = c("black", "aquamarine3"))

# If you want to draw Figure S7 (i.e. simulations without phages)
data_mu6 = filter(data_full, mu == 0)

plot_fitness_nophage = ggplot(data = data_mu6, aes(x = alpha2, y = Fitness, group = alpha2)) +
               geom_point(position = position_jitter(width = 0.05), size = 1) +
               theme_bw(base_size = 7) +
               scale_x_log10() +
               theme(legend.position = "none") +
               xlab("Acquisition probability of competitor") +
               scale_y_continuous(breaks = c(0, 0.2, 0.4, 0.6, 0.8, 1, 1.2)) +
               geom_vline(xintercept = 1e-5, size = 0.2) +
               ylab("Relative fitness")

setwd(Output)
ggsave(plot = plot_fitness_nophage, dpi = 300, file = "FigS7_Revisions.pdf", path = Output, width = 10, height = 6, units = "cm")
```

# 5 Propensity for autoimmunity and optimal CRISPR reactivity

This code can be use to draw Figure 5. To run this code, you need to have run simulations `Compet auto` for different value of propensity (PAM). Each, should be stored in a different folder. Here, I used PAM equals to 0.004, 0.04, 0.4, 4, 40, 400 and 4000.

```
# First we will store the different folders in which the data are stored and then list all their files. Forlders need to contains in their name a string composed of PAM and the value of the propensity (example: "PAM40").

Data0.004 = "/Users/helene/polybox/Postdoc/Research/Acquisition_rate/Submission/Codes/Clean_code_versions/PAM0.004/Data"
Data0.04 = "/Users/helene/polybox/Postdoc/Research/Acquisition_rate/Submission/Codes/Clean_code_versions/PAM0.04/Data"
Data0.4 = "/Users/helene/polybox/Postdoc/Research/Acquisition_rate/Submission/Codes/Clean_code_versions/PAM0.4/Data"
Data4 = "/Users/helene/polybox/Postdoc/Research/Acquisition_rate/Submission/Codes/Clean_code_versions/PAM4/Data"
Data40 = "/Users/helene/polybox/Postdoc/Research/Acquisition_rate/Submission/Codes/Clean_code_versions/PAM40/Data"
Data400 = "/Users/helene/polybox/Postdoc/Research/Acquisition_rate/Submission/Codes/Clean_code_versions/PAM400/Data"
Data4000 = "/Users/helene/polybox/Postdoc/Research/Acquisition_rate/Submission/Codes/Clean_code_versions/PAM4000/Data"

Data = c(Data0.004, Data0.04, Data0.4, Data4, Data40, Data400, Data4000)

liste <-list.files(Data,full.names=T)

data_full <- tibble(alpha1 =c(), alpha2 = c(), mu = c(), t.extinction = c(), t.extinctionB = c(), Prop150_1 = c(), Prop150_2 = c(), Propensity = c())

for (j in levels(factor(liste))){
  data = read_csv(j)
  data <- tibble(alpha1 = data$alpha1, alpha2 = data$alpha2, mu = data$mu, t.extinction = data$t.extinction, t.extinctionB = data$t.extinctionB, Prop150_1 = data$Prop150_1, Prop150_2 = data$Prop150_2)
  extract = str_extract(j, "(?<=PAM)[[:digit:]]+(?=/)") # extract the value of Propensity from the absolute path of the folder. 
  if (is.na(extract) == TRUE){
    extract = str_extract(j, "(?<=PAM)0.[[:digit:]]+(?=/)")
  }
  data <- mutate(data, Propensity = extract)
  data_full = rbind(data_full, data)
}

data_full <- data_full %>%
             mutate(#Propensity = replace(Propensity, is.na(Propensity) == TRUE, 0.4), # correct for the fact that 0.4 is not extracted from the string and therefore results in NA in the dataframe
                    #t.extinction = replace(t.extinction, t.extinction != "Inf", "Yes"),
                    #t.extinction = replace(t.extinction, t.extinction == "Inf", "No"), 
                    #t.extinctionB = replace(t.extinctionB, t.extinctionB != "Inf", "Yes"), 
                    #t.extinctionB = replace(t.extinctionB, t.extinctionB == "Inf", "No"),
                    Fitness = Prop150_2 / (1-Prop150_2)
                    )

# We are only interested in data for which phages can escape CRISPR i.e. when mu = 1e-6.
data_full_evo = filter(data_full, mu == 3.4e-7)

# We want to correct the fitness values in one case: indeed, when both strains went extinct, the fitness equals to NA whereas Fitness should be as 0. simulations where both bacterial strains went extinct:

data_full_evo = mutate(data_full_evo, Fitness = replace(Fitness, is.na(Fitness) == TRUE, 0))

# Now, fitness can range from 0 to Inf. For each condition (Mu*Propensity*Alpha2), we will store in a single dataframe (data_Propensity) the fitness median, its standard deviation and the conditions.

data_Propensity <- data_full_evo %>%
                    group_by(mu, Propensity, alpha2) %>%
                    summarise(Median = median(Fitness))

# Now, we want to select for each propensity, the highest median and store its associated reactivity. 

DataPlot <- data_Propensity %>%
            group_by(Propensity) %>%
            summarise(Reactivity = alpha2[Median == max(Median)])

DataPlot$Propensity = as.numeric(DataPlot$Propensity)

library(scales)
plot.propensity = ggplot(data = DataPlot, aes(x = Propensity, y = Reactivity)) +
                  geom_point(size = 0.5) +
                  geom_line() +
                  ylab("Optimal aquisition") +
                  scale_x_log10(breaks = c(0.004, 0.04, 0.4, 4, 40, 400, 4000), labels = label_number()) +
                  scale_y_log10() +
                  theme_bw(base_size = 12)

ggsave(plot = plot.propensity, file = "Fig5_Revisions.pdf", dpi = 300, path = Output, width = 19.05, height = 9.5, units = "cm")
```

# 6 Competition between type I/II and type III CRISPR-Cas systems

This code can be used to draw panels A and B of Figure 6. For this, you need to have run the simulations `IIIvsIno_auto` (panel A) and `IIIvsI_auto` (panel B) of the Simulations code.

```
# Data auto
Data = ""

# Data No_auto
Data = ""

Output = ""

setwd(Data)

liste <-list.files(Data,full.names=T)

data_compet_type = tibble(alpha1 =c(), alpha2 = c(), mu = c(), Prop150_ST1 = c(), Prop150_ST2 = c()) # required dataset for analysis.

# Here, we charge the data and save everything in a single dataset.
for (j in levels(factor(liste))){
  data = read_csv(j)
  data <- tibble(alpha1 = data$alpha1, alpha2 = data$alpha2, mu = data$mu, Prop150_ST1 = data$Prop150_ST1, Prop150_ST2 = data$Prop150_ST2)
      data_compet_type = rbind(data_compet_type, data)
    }

data_compet_type <- data_compet_type %>%
                    mutate(Cat = Prop150_ST1 > 0.5 ) %>%
                    mutate(Cat = replace(Cat, is.na(Cat) == TRUE, "Ext"))


data_plot_type =  data_compet_type %>%
                  group_by(alpha1, alpha2) %>%
                  summarise(Extinction = sum(Cat == "Ext"), "Selection for type I/II" = sum(Cat == TRUE), "Selection for type III" = sum(Cat == FALSE))
                  

data_plot = data_plot_type %>%
            pivot_longer(c("Extinction", "Selection for type I/II", "Selection for type III"), names_to = "outcome", values_to = "count") %>%
            group_by(alpha1, alpha2) %>%
            slice_max(count)

plotIIIvsI_auto <- ggplot(data = data_plot, aes(x = alpha1, y = alpha2, fill = outcome)) +
              geom_tile(color = "white", lwd = 1, linetype = 1) +
              geom_text(aes(label = round(count, 1)), colour = "white", size = 1) +
              coord_fixed() +
              geom_abline(intercept = 0, slope = 1) +
              theme_bw(base_size = 7) +
              theme(legend.position = "bottom", plot.subtitle = element_text(size = 7), plot.title = element_text(size = 7)) +
               xlab("Spacer acquisition of type I/II CRISPR-Cas system") +
               ylab("Spacer acquisition of type III CRISPR-Cas system") +
               scale_x_log10() +
               scale_y_log10() +
               scale_fill_discrete(name = "Most frequent outcome")
               #scale_fill_gradient2(low = "deepskyblue", mid = "gainsboro", high = "firebrick1", midpoint = 0.5)
               scale_fill_c
```

# 7 Patchwork codes

Use these codes to combine several panels in one figure.

```
Fig3 = plot_No_auto / (plot_proba + plot.div) + plot_annotation(tag_levels = 'A')

ggsave(plot = Fig3, dpi = 300, file = "Figure3Revisions.pdf", path = Output, width = 19.05, height =18, units = "cm")

Fig4 = plot_fitness_no_auto + plot_fitness_auto + plot_annotation(tag_levels = 'A')
ggsave(plot = Fig4, dpi = 300, file = "Figure4_Revisions.pdf", path = Output, width = 19.05, height = 9.5, units = "cm")

fig6 = plotIIIvsI_no_auto + plotIIIvsI_auto + plot_layout(guides = 'collect') + plot_annotation(tag_levels = 'A') & theme(legend.position = "bottom")

ggsave(fig6, file = "Fig6_Revisions.pdf", dpi = 300, path = Output, width = 19.05, height = 9.5, units = "cm")

FigS4 = plot_PR190 + plot_PR19 + plot_annotation(tag_levels = 'A') + plot_layout(guides = 'collect') & theme(legend.position = "bottom")

ggsave(plot = FigS4, dpi = 300, file = "FigureS4_Revisions.pdf", path = Output, width = 19.05, height = 9.5, units = "cm")

layout <- "
AE
BF
CG
DH
"

FigS10 = plot_beta2 + plot_beta3 + plot_beta4 + plot_beta5 + plot_beta6 + plot_beta7 + plot_beta8 + guide_area() + plot_annotation(tag_levels = 'A') + plot_layout(design = layout, guides = 'collect')

ggsave(plot = FigS10, dpi = 300, file = "FigureS10_Revisions.pdf", path = Output, width = 19.05, height = 22.23, units = "cm")
```
