# Supplementary material for: Epidemiological and evolutionary consequences of different types of CRISPR-Cas systems
Source: PLoS Comput Biol. 2022 Jul 26;18(7):e1010329. doi: 10.1371/journal.pcbi.1010329 (PMC9355216; doi:10.1371/journal.pcbi.1010329)
Supplement: S1 Codes — (ZIP) [file pcbi.1010329.s001.zip › acquisition-main/Simulations.html]

Simulations: Epidemiological and evolutionary consequences of different types of CRISPR-Cas systems


Code 

- Show All Code
- Hide All Code

# Simulations: Epidemiological and evolutionary consequences of different types of CRISPR-Cas systems

#### Hélène Chabas, Viktor Müller, Sebastian Bonhoeffer and Roland Regoes

#### January 27, 2022

# 1 Packages, source and folders

## 1.1 Required Packages

These packages need to have been preinstalled before being used.

```
library(adaptivetau)
library(ggplot2)
library(ggthemes)
library(pracma)
library(doParallel)
library(dplyr)
library(gridExtra)
```

## 1.2 Folders and parallelisation.

```
## Please add the absolute path of the Models.R file.
source("")

## Please add the absolute path of the folder you want the data resulting from simulations to be stored.
Data = ""

## To make the simulations faster, the code is partially parallalised. Please add the number of cores of your computer you want to use for the simulations.
registerDoParallel(cores = 5)
```

# 2 CRISPR Reactivity and epidemiological outcome

## 2.1 No autoimmunity

These simulations runs for an initial conditions with no resistant bacteria, no escape phage mutants. Phages starts infecting bacteria at a MOI of 0.01.

There is different parameters: - burstWT: number of WT virus progeny - burstPR: number of escape mutants progeny - g: vector that contains the growth rate of first the sensitive bacteria then the resistant. - ns: maximum number of spacers - alpha: CRISPR Reactivity (Probability to acquire a spacer) - mu: phage protospacer mutation probability - beta: infectivity - K: carrying capacity

We will vary 1) CRISPR Reactivity and 2) phage mutation probability. For each of the simulation, we will: - calculate Nei diversity - determine if and when the phage goes extinct - calculate the size of the epidemic (total number of virions produced). - If we want, we can also draw time courses of each simulation.

We run 100 simulations per condition. We save the values of parameters in csv files.

```
# Required vectors and dataframes
Size.epidemics <- c()
alpha <- c()
t.extinction <- c()
t.extinctionB <- c()
alpha1 = c()
mu1 = c()
mu = c()
burstPR1 = c()
burstWT1 = c()
Nei_i = c()
Nei_f = c()

data_Nei2 <- data.frame(NeiR = c(), NeiPR =  c(), D1R = c(), D1P =c(), Prop_bacR = c(), Prop_PR = c(), time = c(), alpha = c(), mu = c(), TotPhage = c(), Tot_bac =c(), BWT = c(), PWT = c(), TotBIM = c(), TotPR = c())

j = 1e-6 # Please, set j with the protospacer probability you want to use in your simulations

# Simulations
result <- foreach(i = 1:100) %dopar% { # i represents the number of simulations that will be run, here 100.
    params <- list(burstWT = 100, burstPR = 70, g = c(1.3, 1.3), ns = 100, alpha = 1e-8, mu = j, beta = 1e-6, K = 1e11)
    while (params$alpha < 1e-1){
        output <- model.multi.evo(params)
        outputR <- output[2:(params$ns +2)] # output for bacteria including S
        outputPR <- output[(params$ns+3) : (2*params$ns+3)] # output for phage including WT

        # Calcul of 1D Diversity for both phages and bacteria
        D1P = apply(outputPR != 0, 1, sum) # phages
        D1R = apply(outputR != 0, 1, sum) # bacteria

        # Calcul of Nei diversity
        SommeR <- apply(outputR, 1, sum) # Calcul of the sum of each line. Total of bacteria including S.
        SommePR <- apply(outputPR, 1, sum) # Idem for phages

        # Calcul of the proportion of each genotype.
        OutputR1 <- outputR / SommeR  #Bacteria
        OutputPR1 <- outputPR / SommePR #Phages

        # Calcul of the proportion^2
        OutputR2 <- OutputR1 ^2  #Bacteria
        OutputPR2 <- OutputPR1 ^2 #Phages

        # Calcul of the sum of pi^2
        SommeR1 <- apply(OutputR2, 1, sum) # Bacteria
        SommePR1 <- apply(OutputPR2, 1, sum) # Phages

        # Calcul of Genetic diversity
        NeiR <- 1/SommeR1 # Bacteria
        NeiPR <- 1/SommePR1 # Phages

        Prop_bacR <- 1 - OutputR1$S # Proportion of resistant bacteria in the population.
        Prop_PR <- 1 - OutputPR1$P # Proportion of escape phages in the population.

        # We will store the values of parameters that can vary.
        mu <- rep(params$mu, length(NeiR))
        alpha <- rep(params$alpha, length(NeiR))
        burstPR <- rep(params$burstPR, length(NeiR))

        TotBIM = SommeR - output$S # Total number of BIM bacteria
        TotPR = SommePR - output$P # Total number of escape mutants

        data_Nei <- data.frame(NeiR, NeiPR, D1R, D1P, Prop_bacR, Prop_PR, time = output$time, alpha, mu, TotPhage = SommePR, Tot_bac = SommeR, BWT = output$S, PWT = output$P, TotBIM, TotPR)
        data_Nei2 <- rbind(data_Nei2, data_Nei)

        # If you want to plot time courses for some values of alpha, please uncomment the following code. Please note that this makes the simulations running for longer and that this requires storage space.

        #if (params$alpha == 5.12e-06){

        ## Time courses for phage and bacteria
        #plot.phage = ggplot() +
        #                geom_line(data = data_Nei, aes(x = time, y = PWT), colour = "black") +
        #                geom_line(data = data_Nei, aes(x = time, y = TotPR), colour = "purple3") +
        #                theme_bw(base_size = 15) +
        #                ylab("Phages") +
        #                xlab("Time")

        #plot.bacteria = ggplot() + 
        #                geom_line(data = data_Nei, aes(x = time, y = BWT), colour = "black") +
        #                geom_line(data = data_Nei, aes(x = time, y = TotBIM), colour = "cyan3") +
        #                theme_bw(base_size = 15) +
        #                ylab("Bacteria") +
        #                xlab("Time")

        #ggsave(plot = plot.phage, file = paste("plot_phage_alpha", params$alpha, "_mu", params$mu, "_", i, ".pdf"), width = 10, height = 7, units = "cm", dpi = 300)
        #ggsave(plot = plot.bacteria, file = paste("plot_bacteria_alpha", params$alpha, "_mu", params$mu, "_", i, ".pdf"), width = 10, height = 7, units = "cm", dpi = 300)

        ## Stack plots for phages and bacteria.

        #outputR$time = output$time

        #long_outputB = gather(data = outputR, key = BIM, value = number, ... = R1:S)

        #plot.bac.stack = ggplot(data = long_outputB, aes(x = time, y = number, fill = BIM), colour = "black") +
        #                geom_area(position = 'stack') +
        #                theme_bw(base_size = 15) +
        #                theme(legend.position = "none") +
        #                ylab("Bacteria") +
        #                 xlab("Time")

        #ggsave(plot = plot.bac.stack, file = paste("plot_bac_stack_alpha", params$alpha, "_mu", params$mu, "_", i, ".pdf"), width = 10, height = 7, units = "cm", dpi = 300)

        #outputPR$time = output$time

        #long_outputPR = gather(data = outputPR, key = PIM, value = number, ... = P:PR100)

        #plot.phage.stack = ggplot(data = long_outputPR, aes(x = time, y = number, fill = PIM)) +
        #                geom_area(position = 'stack') +
        #                theme_bw(base_size = 15) +
        #                theme(legend.position = "none") +
        #                ylab("Phage") +
        #                xlab("Time")

        #ggsave(plot = plot.phage.stack, file = paste("plot_phage_stack_alpha", params$alpha, "_mu", params$mu, "_", i, ".pdf"), width = 10, height = 7, units = "cm", dpi = 300)

        #}

        ## Extinction data

        # Here, we will store some parameters
        alpha1 = c(alpha1, params$alpha)
        mu1 <- c(mu1, params$mu)
        burstPR1 = c(burstPR1, params$burstPR)
        burstWT1 = c(burstWT1, params$burstWT)

        # Here, we will store the time of phage extinction (t.extinction) and the time for bacterial extinction (t.extinctionB) and the size of the epidemics (Size.epidemics)
        t.extinction <- c(t.extinction, min(data_Nei$time[data_Nei$TotPhage == 0 & data_Nei$time > data_Nei$time[data_Nei$TotPhage == max(data_Nei$TotPhage)]]))
        t.extinctionB <- c(t.extinctionB, min(data_Nei$time[data_Nei$Tot_bac == 0 & data_Nei$time > data_Nei$time[data_Nei$Tot_bac == max(data_Nei$Tot_bac)]]))
        Size.epidemics = c(Size.epidemics, trapz(x = data_Nei$time, y = data_Nei$TotPhage))

        # We want to store Nei diversity at the beginning of the outbreak (i.e. when sensitive bacteria go extinct) and at the end of the simulations (time = 150)
        Nei_i = c(Nei_i, min(data_Nei$NeiR[data_Nei$time == min(data_Nei$time[data_Nei$BWT == 0])]))
        Nei_f = c(Nei_f, data_Nei$NeiR[data_Nei$time == 150])

        params$alpha <- 2 * params$alpha
        }
    data_Size = data.frame(alpha1, mu1, burstPR1, burstWT1, t.extinction, t.extinctionB, Size.epidemics, Nei_i, Nei_f)
    return(data_Size)
    }
data_Size = bind_rows(result, .id = "column_label")
write.csv(data_Size, file = paste0("Size_Mu", j, ".csv")) # Saving data for data_Size
```

## 2.2 Autoimmunity

These simulations work exactly as the one above, the only difference is that they are not calling the same model.

This code is required to generate data to make Figures S6 and S8. In params, PAM = 40 for Figure S6 and PAM = 4000 for Figure S8.

```
Data = "" # Absolute path of the folder where you want to store the data.
setwd(Data)

# Empty vectors and dataframes 
Size.epidemics <- c()
alpha <- c()
t.extinction <- c()
t.extinctionB <- c()
alpha1 = c()
mu1 = c()
mu = c()
burstPR1 = c()
burstWT1 = c()

data_Nei2 <- data.frame(NeiR = c(), NeiPR =  c(), D1R = c(), D1P =c(), Prop_bacR = c(), Prop_PR = c(), time = c(), alpha = c(), mu = c(), TotPhage = c(), Tot_bac =c(), BWT = c(), PWT = c(), TotBIM = c(), TotPR = c())

j = 1e-6 # Please, set j with the protospacer probability you want to use in your simulations

result <- foreach(i = 1:100) %dopar% { # i = number of simulations
    params <- list(burstWT = 100, burstPR = 70, g = c(1.3, 1.3), ns = 100, alpha = 1e-8, mu = j, beta = 1e-6, K = 1e11, self = 1, PAM = 40)
    while (params$alpha < 1e-1){
        output <- model.auto(params)
        outputR <- output[2:(params$ns +2)] # output for bacteria including S
        outputPR <- output[(params$ns+3) : (2*params$ns+3)] # output for phage

        #Calcul of 1D Diversity for both phages and bacteria
        D1P = apply(outputPR != 0, 1, sum) # phages
        D1R = apply(outputR != 0, 1, sum) # bacteria

        # Calcul of the Nei diversity
        SommeR <- apply(outputR, 1, sum) # Calcul of the sum of each line. Total of bacteria including S.
        SommePR <- apply(outputPR, 1, sum)

        OutputR1 <- outputR / SommeR # Calcul of the proportion of each genotype
        OutputPR1 <- outputPR / SommePR

        OutputR2 <- OutputR1 ^2 # Calcul of the proportion^2
        OutputPR2 <- OutputPR1 ^2

        SommeR1 <- apply(OutputR2, 1, sum) # Calcul of the sum of pi^2
        SommePR1 <- apply(OutputPR2, 1, sum)

        NeiR <- 1/SommeR1 #Calcul of Genetic diversity
        NeiPR <- 1/SommePR1

        Prop_bacR <- 1 - OutputR1$S # Proportion of resistant bacteria in the population.
        Prop_PR <- 1 - OutputPR1$P # Proportion of escape phages in the population.

        mu <- rep(params$mu, length(NeiR))
        alpha <- rep(params$alpha, length(NeiR))
        burstPR <- rep(params$burstPR, length(NeiR))

        TotBIM = SommeR - output$S # Total number of BIM bacteria
        TotPR = SommePR - output$P # Totla number of escape mutants

        data_Nei <- data.frame(NeiR, NeiPR, D1R, D1P, Prop_bacR, Prop_PR, time = output$time, alpha, mu, TotPhage = SommePR, Tot_bac = SommeR, BWT = output$S, PWT = output$P, TotBIM, TotPR)
        data_Nei2 <- rbind(data_Nei2, data_Nei)

        #If you want to plot the epidemic for each simulation, insert this in the loop. Cautious, this increases the simulation time.

        #plot.epidemic = ggplot() +
                  #geom_line(data = data_Nei, aes(x = time, y = BWT), colour = "blue") +
                  #geom_line(data = data_Nei, aes(x = time, y = TotBIM), colour = "cyan3") +
                  #geom_line(data = data_Nei, aes(x = time, y = PWT), colour = "red") +
                  #geom_line(data = data_Nei, aes(x = time, y = TotPR), colour = "brown3") +
                  #theme_bw(base_size = 30) +
                  #scale_y_log10() +
                  #ylab("Phage/Bacteria") +
                  ##theme(legend.position = "top") +
                  #xlab("Time")

        #ggsave(plot = plot.epidemic, file = paste("plot_epidemic_alpha", params$alpha, "_mu", params$mu, "_", i, ".pdf"))

        alpha1 = c(alpha1, params$alpha)
        mu1 <- c(mu1, params$mu)
        burstPR1 = c(burstPR1, params$burstPR)
        burstWT1 = c(burstWT1, params$burstWT)

        t.extinction <- c(t.extinction, min(data_Nei$time[data_Nei$TotPhage == 0 & data_Nei$time > data_Nei$time[data_Nei$TotPhage == max(data_Nei$TotPhage)]]))
        t.extinctionB <- c(t.extinctionB, min(data_Nei$time[data_Nei$Tot_bac == 0 & data_Nei$time > data_Nei$time[data_Nei$Tot_bac == max(data_Nei$Tot_bac)]]))
        Size.epidemics = c(Size.epidemics, trapz(x = data_Nei$time, y = data_Nei$TotPhage))

        params$alpha <- 2 * params$alpha
        }
    data_Size = data.frame(alpha1, mu1, burstPR1, burstWT1, t.extinction, t.extinctionB, Size.epidemics)
    write.csv(data_Nei2, file = paste0("Nei_Mu", j, "_", i, ".csv"))
    return(data_Size)
    }
data_Size = bind_rows(result, .id = "column_label")
write.csv(data_Size, file = paste0("Size_Mu", j, ".csv")) # Saving data for data_Size
```

# 3 Initial bacterial diversity and probability of phage extinction.

These simulations are required to make figure S1.

```
setwd(Data)

Size.epidemics <- c()
alpha <- c()
t.extinction <- c()
alpha1 = c()
mu1 = c()
mu = c()
burstPR1 = c()
burstWT1 = c()
div = c()

data_Nei2 <- data.frame(NeiR = c(), NeiPR =  c(), time = c(), alpha = c(), mu = c())

for (k in c(1, 2, 4, 6, 8, 10, 12, 14, 16, 18, 20, 22, 24, 48)){ # k initial diversity of resistant bacteria
   result <- foreach(i = 1:100) %dopar% { # i = number of simulations
      params <- list(burstWT = 190, burstPR = 179, g = c(0.44, 0.44), ns = 100, div = k, alpha = 0, mu = 3.4e-7, beta = 1e-6, K = 1e11)
      output <- model.div.multi.evo(params)
      outputR <- output[2:(params$ns +2)] # output for bacteria
      outputPR <- output[(params$ns+3) : (2*params$ns+3)] # output for phage

        # Calcul of the Nei diversity

      SommeR <- apply(outputR, 1, sum) # Calcul of the sum of each line
      SommePR <- apply(outputPR, 1, sum)

      OutputR1 <- outputR / SommeR # Calcul of the proportion
      OutputPR1 <- outputPR / SommePR

      OutputR2 <- OutputR1 ^2 # Calcul of the proportion^2
      OutputPR2 <- OutputPR1 ^2

      SommeR1 <- apply(OutputR2, 1, sum) # Calcul of the sum of pi^2
      SommePR1 <- apply(OutputPR2, 1, sum)

      NeiR <- 1/SommeR1 #Calcul of Genetic diversity
      NeiPR <- 1/SommePR1

      mu <- rep(params$mu, length(NeiR))
      alpha <- rep(params$alpha, length(NeiR))
      burstPR <- rep(params$burstPR, length(NeiR))

      data_Nei <- data.frame(NeiR, NeiPR, time = output$time, alpha, mu, SommePR, SommeR)
                 #data_Nei2 <- rbind(data_Nei2, data_Nei)

        alpha1 = c(alpha1, params$alpha)
      mu1 <- c(mu1, params$mu)
      burstPR1 = c(burstPR1, params$burstPR)
      burstWT1 = c(burstWT1, params$burstWT)
      div = c(div, params$div)
      t.extinction <- c(t.extinction, min(data_Nei$time[data_Nei$SommePR == 0 & data_Nei$time > data_Nei$time[data_Nei$SommePR == max(data_Nei$SommePR)]]))
      Size.epidemics = c(Size.epidemics, trapz(x = data_Nei$time, y = data_Nei$SommePR))
      data_Size = data.frame(alpha1, mu1, burstPR1, burstWT1, div, t.extinction, Size.epidemics)
      return(data_Size)
      }
  data_Size = bind_rows(result, .id = "column_label")
  write.csv(data_Size, file = paste0("Div",k,"_bact_Size_Mu34e-7.csv")) # Saving data for data_Size }
}
```

# 4 Simulations of competitions

## 4.1 No autoimmunity

This code is required to simulate data for Figure 4, panel A.

```
# Add the absolute path of the folder where you want to store the data resulting from the simulations.

Data = ""
setwd(Data)

# Here, we create empty vectors and dataframes we need for the simulations.
Size.epidemics <- c()
t.extinction <- c()
t.extinctionB <- c()

Prop150_1 = c()
Prop150_2 = c()
alphab2 = c()
alphab1 = c()
mu1 = c()
Mut = c()

num =  # Please add the value of the CRISPR Reactivity of second strain.

for (j in c(0, 1e-6)){
  result <- foreach(i = 1:100) %dopar% {
     params <- list(burstWT = 100, burstPR = 70, g = c(1.3, 1.3), ns = 100, alpha1 = 1e-5, alpha2 = num, mu = j, beta = 1e-6, K = 1e11, self = 1, PAM = 40)
           output <- model.compet(params)
           outputS1 <- output[2:(params$ns +2)] # output for bacteria R and S1
           outputS2 <- output[(2*(params$ns+1)+2):((3*(params$ns+1))+1)] # output for bacteria S2 and resistant bacteria
           outputPR <- output[(params$ns+3) : (2*params$ns+3)] # output for phage

           #With this data, we try to answer one question: what is the relative fitness of the two strains?

           # So we need to calculate: the frequency of one strain at the end (at t0 it is 0.5). We also store extinction of bacteria and phages.

           # Lets start to calculate the proportion of S+resistant bacteria for each strain

           Total = apply(outputS1, 1, sum) + apply(outputS2, 1, sum) # Total number of bacteria
           Prop1 = apply(outputS1, 1, sum) / Total # Proportion of bacteria 1
           Prop2 = apply(outputS2, 1, sum) / Total # Proportion of bacteria 2
           
           #Then, we store it in a data frame
           Prop = data.frame(Time = output$time, Prop1, Prop2, Total)

           # Here, we store the proportions of the two strains at the end of the simulation.
           Prop150_1 = c(Prop150_1, Prop$Prop1[Prop$Time == 150]) 
           Prop150_2 = c(Prop150_2, Prop$Prop2[Prop$Time == 150])

           # Now, let's save the data of time extinction
           alphab1 = c(alphab1, params$alpha1)
           alphab2 = c(alphab2, params$alpha2)
           mu1 <- c(mu1, params$mu)

           t.extinction <- c(t.extinction, min(Prop$Time[Prop$TotPhage == 0 & Prop$Time > Prop$Time[Prop$TotPhage == max(Prop$TotPhage)]]))
           t.extinctionB <- c(t.extinctionB, min(Prop$Time[Prop$Total == 0 & Prop$Time > Prop$Time[Prop$Total == max(Prop$Total)]]))

           data_Size = data.frame(alpha1 = alphab1, alpha2 = alphab2, mu = mu1, t.extinction, t.extinctionB, Prop150_1, Prop150_2)
          return(data_Size)
         }
data_Size = bind_rows(result)
write.csv(data_Size, file = paste0("Size_Mu", j,"_", num, ".csv"))
}
```

## 4.2 Autoimmunity

These simulations are coded in the same way as the one above. They call a different model that is taking into account autoimmunity.

This code is required to generate data to draw Figure 4, panel B. This code is also required to draw all the panels of figure S10. To do so, you need to run this code with various values of `params$beta` (see Sup Info).

Finally, you also need this code to draw Figure 5 with PAM equals to 0.4, 4, 40, 400 and 4000.

```
# Empty vectors
Size.epidemics <- c()
t.extinction <- c()
t.extinctionB <- c()
Prop150_1 = c()
Prop150_2 = c()
alphab2 = c()
alphab1 = c()
mu1 = c()
Mut = c()

num = 1e-7 # Please, change num to run competitions with various CRISPR Reactivity for one of the strain.

for (j in c(0, 1e-6)){ # j: Protospacer mutation probability
  result <- foreach(i = 1:100) %dopar% { # i = number of simulations
           params <- list(burstWT = 100, burstPR = 70, g = c(1.3, 1.3), ns = 100, alpha1 = 1e-5, alpha2 = num, mu = j, beta = 1e-6, K = 1e9, self = 1, PAM = 40)
           output <- model.compet.auto(params)
           outputS1 <- output[2:(params$ns +2)] # output for bacteria R and S1
           outputS2 <- output[(2*(params$ns+1)+2):((3*(params$ns+1))+1)] # output for bacteria S2 and BR
           outputPR <- output[(params$ns+3) : (2*params$ns+3)] # output for phage

           #With this data, we try to answer this questions: what is the fitness of the two strains?

           # So we need to calculate the frequency of one strain at the end (at t0 it is 50%)

           # Lets start to calculate the proportion of S+BR
           Total = apply(outputS1, 1, sum) + apply(outputS2, 1, sum)
           Prop1 = apply(outputS1, 1, sum) / Total
           Prop2 = apply(outputS2, 1, sum) / Total

           Prop = data.frame(Time = output$time, Prop1, Prop2, Total)

           # Let's separate the values of each proportion at the end of the simulation.
           Prop150_1 = c(Prop150_1, Prop$Prop1[Prop$Time == 150])
           Prop150_2 = c(Prop150_2, Prop$Prop2[Prop$Time == 150])

           # Now, let's save the data of time extinction

           alphab1 = c(alphab1, params$alpha1)
           alphab2 = c(alphab2, params$alpha2)
           mu1 <- c(mu1, params$mu)

           data_Size = data.frame(alpha1 = alphab1, alpha2 = alphab2, mu = mu1, Prop150_1, Prop150_2)
          return(data_Size)
         }
data_Size = bind_rows(result)
write.csv(data_Size, file = paste0("Size_Mu", j,"_", num, ".csv"))
}
```

## 4.3 In absence of phages

You need to run these simulations to generate data for Figure S7.

```
Size.epidemics <- c()
t.extinction <- c()
t.extinctionB <- c()

Prop150_1 = c()
Prop150_2 = c()
alphab2 = c()
alphab1 = c()
mu1 = c()
Mut = c()

for (j in c(0, 1e-6)){
  result <- foreach(i = 1:100) %dopar% {
     params <- list(burstWT = 100, burstPR = 70, g = c(1.3, 1.3), ns = 100, alpha1 = 1e-5, alpha2 = num, mu = j, beta = 1e-6, K = 1e11, self = 1, PAM = 40)
           output <- model.compet.auto.nophage(params)
           outputS1 <- output[2:(params$ns +2)] # output for bacteria R and S1
           outputS2 <- output[(2*(params$ns+1)+2):((3*(params$ns+1))+1)] # output for bacteria S2 and BR
           outputPR <- output[(params$ns+3) : (2*params$ns+3)] # output for phage

           #With this data, we try to answer two questions: what is the fitness of the two strains? Which one drive the dynamic?

           # So we need to calculate: the frequency of one strain at the end (at t0 it is 50%) and to store the extinction of bacteria and phages.

           # Lets start to calculate the proportion of S+BR

           Total = apply(outputS1, 1, sum) + apply(outputS2, 1, sum)
           Prop1 = apply(outputS1, 1, sum) / Total
           Prop2 = apply(outputS2, 1, sum) / Total
           PropR1 = apply(outputS1, 1, sum) - outputS1$S1 
           PropR2 = apply(outputS2, 1, sum) - outputS2$S2
           D1R1 = apply(outputS1 != 0, 1, sum) # number of genotypes bacteria 1
           D1R2 = apply(outputS2 != 0, 1, sum) # number of genotypes bacteria 2

           TotPhage = apply(outputPR, 1, sum)

           #Then, we store it in a data frame

           Prop = data.frame(Time = output$time, Prop1, Prop2, PropR1, PropR2, TotPhage, Total, D1R1, D1R2)
           #write.csv(Prop, file = paste0("Prop", j,"_",params$alpha2, "_", i, ".csv"))

           Prop150_1 = c(Prop150_1, Prop$Prop1[Prop$Time == 150])
           Prop150_2 = c(Prop150_2, Prop$Prop2[Prop$Time == 150])

           # Now, let's save the data of time extinction

           alphab1 = c(alphab1, params$alpha1)
           alphab2 = c(alphab2, params$alpha2)
           mu1 <- c(mu1, params$mu)

           t.extinction <- c(t.extinction, min(Prop$Time[Prop$TotPhage == 0 & Prop$Time > Prop$Time[Prop$TotPhage == max(Prop$TotPhage)]]))
           t.extinctionB <- c(t.extinctionB, min(Prop$Time[Prop$Total == 0 & Prop$Time > Prop$Time[Prop$Total == max(Prop$Total)]]))

           data_Size = data.frame(alpha1 = alphab1, alpha2 = alphab2, mu = mu1, t.extinction, t.extinctionB, Prop150_1, Prop150_2)
          return(data_Size)
         }
data_Size = bind_rows(result)
write.csv(data_Size, file = paste0("Size_Mu", j,"_", num, ".csv"))
}
```

# 5 Competition between type III and type I/II CRISPR-Cas systems

## 5.1 No autoimmunity

```
Size.epidemics <- c()
t.extinction <- c()
t.extinctionB <- c()
Prop150_ST1 = c()
Prop150_ST2 = c()

alphab2 = c()
alphab1 = c()
mu1 = c()
Mut = c()
j = 3.4e-7

#num = 1e-7 # Please, change num to run competitions with various CRISPR Reactivity for one of the strain.

result <- foreach(i = 1:100) %dopar% { # i = number of simulations
    params <- list(burstWT = 190, burstPR = 179, g = c(0.44, 0.44), ns = 100, alpha1 = num1, alpha2 = num2, mu = j, beta = 1e-6, K = 1e11, self = 1, PAM = 40)
    output <- model.compet.noauto.IIIvsI(params)
    outputST1 <- select(output, R1:paste("R", params$ns, sep =""), S1) # output for bacteria R and S1
    outputST2 <- select(output, BR1:paste("BR", params$ns, sep =""), S2) # output for bacteria BR

    #With this data, we try to answer this questions: what is the fitness of the two strains?

    # So we need to calculate the frequency of one strain at the end (at t0 it is 50%)

    # Lets start to calculate the proportion of S+BR
    Total = apply(outputST1, 1, sum) + apply(outputST2, 1, sum)
    PropST1 = apply(outputST1, 1, sum) / Total # Proportion of Strain 1
    PropST2 = apply(outputST2, 1, sum) / Total # Proportion of Strain 2

    Prop = data.frame(Time = output$time, Prop_ST1 = PropST1, Prop_ST2 = PropST2, Total = Total)

    # Let's separate the values of each proportion at the end of the simulation.
    Prop150_ST1 = c(Prop150_ST1, Prop$Prop_ST1[Prop$Time == 150])
    Prop150_ST2 = c(Prop150_ST2, Prop$Prop_ST2[Prop$Time == 150])

    # Now, let's save the data of time extinction

    alphab1 = c(alphab1, params$alpha1)
    alphab2 = c(alphab2, params$alpha2)
    mu1 <- c(mu1, params$mu)

    data_Size = data.frame(alpha1 = alphab1, alpha2 = alphab2, mu = mu1, Prop150_ST1, Prop150_ST2)
    return(data_Size)
    }
data_Size = bind_rows(result)
write.csv(data_Size, file = paste0("CompetIIIvsI_", num1, "_", num2, ".csv"))
```

## 5.2 Autoimmunity

```
Size.epidemics <- c()
t.extinction <- c()
t.extinctionB <- c()
Prop150_ST1 = c()
Prop150_ST2 = c()

alphab2 = c()
alphab1 = c()
mu1 = c()
Mut = c()
j = 3.4e-7

#num = 1e-7 # Please, change num to run competitions with various CRISPR Reactivity for one of the strain.

result <- foreach(i = 1:100) %dopar% { # i = number of simulations
    params <- list(burstWT = 190, burstPR = 179, g = c(0.44, 0.44), ns = 100, alpha1 = num1, alpha2 = num2, mu = j, beta = 1e-6, K = 1e11, self = 1, PAM = 40)
    output <- model.compet.auto.IIIvsI(params)
    outputST1 <- select(output, R1:paste("R", params$ns, sep =""), S1) # output for bacteria R and S1
    outputST2 <- select(output, BR1:paste("BR", params$ns, sep =""), S2) # output for bacteria BR

    #With this data, we try to answer this questions: what is the fitness of the two strains?

    # So we need to calculate the frequency of one strain at the end (at t0 it is 50%)

    # Lets start to calculate the proportion of S+BR
    Total = apply(outputST1, 1, sum) + apply(outputST2, 1, sum)
    PropST1 = apply(outputST1, 1, sum) / Total # Proportion of Strain 1
    PropST2 = apply(outputST2, 1, sum) / Total # Proportion of Strain 2

    Prop = data.frame(Time = output$time, Prop_ST1 = PropST1, Prop_ST2 = PropST2, Total = Total)

    # Let's separate the values of each proportion at the end of the simulation.
    Prop150_ST1 = c(Prop150_ST1, Prop$Prop_ST1[Prop$Time == 150])
    Prop150_ST2 = c(Prop150_ST2, Prop$Prop_ST2[Prop$Time == 150])

    # Now, let's save the data of time extinction

    alphab1 = c(alphab1, params$alpha1)
    alphab2 = c(alphab2, params$alpha2)
    mu1 <- c(mu1, params$mu)

    data_Size = data.frame(alpha1 = alphab1, alpha2 = alphab2, mu = mu1, Prop150_ST1, Prop150_ST2)
    return(data_Size)
    }
data_Size = bind_rows(result)
write.csv(data_Size, file = paste0("CompetIIIvsI_", num1, "_", num2, ".csv"))
```
