# Supplementary material for: Epidemiological and evolutionary consequences of different types of CRISPR-Cas systems
Source: PLoS Comput Biol. 2022 Jul 26;18(7):e1010329. doi: 10.1371/journal.pcbi.1010329 (PMC9355216; doi:10.1371/journal.pcbi.1010329)
Supplement: S1 Text — A list of all captions is provided here: Impact of initial population-wide spacer diversity on the probability of phage extinction.The figure shows the probability of phage extinction in 100 simulations. At the beginning of the simulations, the bacterial population was composed of 6*106 bacteria with an equal representation of sensitive bacteria (S) and different numbers of resistant bacterial genotypes. These populations are infected by 105 PWT phages, with phage evolution set to μ = 3.4*10−7 and the probability of spacer acquisition set to α = 0.Influence of CRISPR-Cas probability of spacer acquisition on the mean diversity of newly generated spacers at the beginning (when S goes extinct, panel A) or at the end (panel B) of the outbreak. For simulations resulting in bacterial extinction at the relevant time, we set diversity to 0. We provide the proportion of bacterial extinction at the beginning (panel C) and at the end (panel D) of the simulations.The black curve represents the initial diversity of spacers (when S goes extinct) where phage cannot evolve (μ = 0) and the purple, green and red curves when phages can evolve (μ = 10−8, μ = 3.4*10−7, μ = 10−6 respectively). On Panel B, the grey line represents the initial diversity. Error bars correspond to 95% confidence intervals and are barely visible due to limited variation.Influence of phage infectivity on the epidemiological outcome in the absence of autoimmunity.Probability of phage survival when infecting bacteria with various probabilities of spacer acquisition. The different colors correspond to different levels of phage evolution (μ): in black, no evolution type III CRISPR-Cas system (μ = 0), in purple μ = 10−8; in green type I/II CRISPR-Cas system μ = 3.4*10−7 and in red μ = 10−6. The different panels represent various phage infectivity: A) β = 10−2, B) β = 10−3, C) β = 10−4, D) β = 10−5, E) β = 10−6, F) β = 10−7, G) β = 10−8.Probability of survival, for a phage infecting bacteria with various probabiliti [file pcbi.1010329.s002.pdf]

Supplementary informations – Epidemiological and Evolutionary Consequences of different types of CRISPR-Cas Systems

Hélène Chabas[1], Viktor Müller[2], Sebastian Bonhoeffer[1*] & Roland Regoes[1*]

[1] Institute for Integrative Biology, ETH Zürich, Switzerland

[2] Institute of Biology, Eötvös Loránd University, Budapest, Hungary

[*] Equal contributions

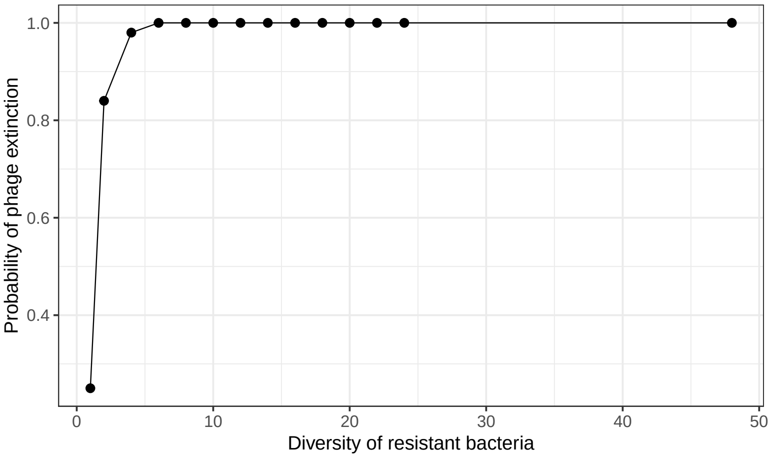

Figure A: Impact of initial population-wide spacer diversity on the probability of phage extinction.

The figure shows the probability of phage extinction in 100 simulations. At the beginning of the simulations, the bacterial population was composed of $6 * 10^6$ bacteria with an equal representation of sensitive bacteria (S) and different numbers of resistant bacterial genotypes. These populations are infected by 10^5 P_{WT} phages, with phage evolution set to $\mu = 3.4 * 10^{-7}$ and the probability of spacer acquisition set to $\alpha = 0$.

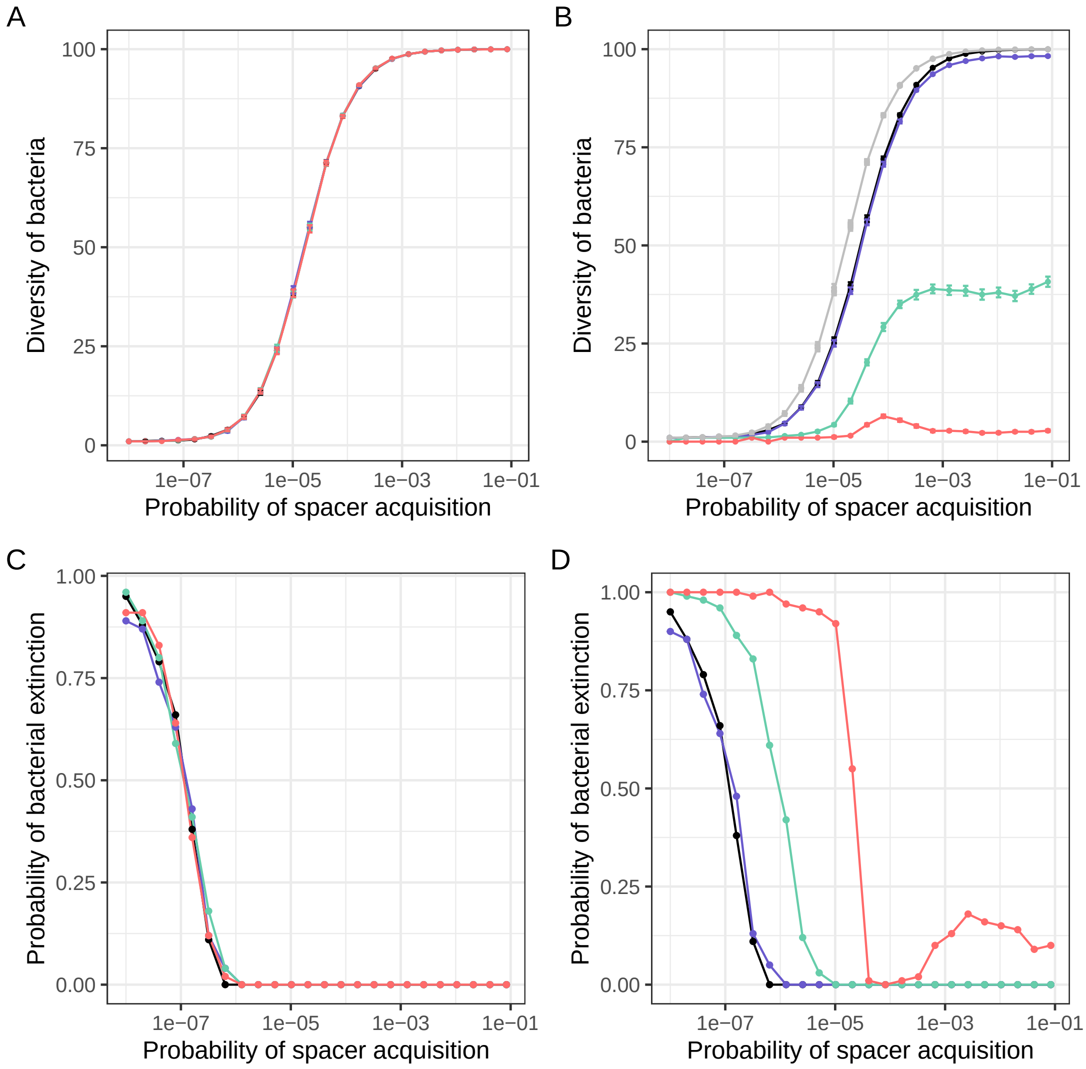

Figure B: Influence of CRISPR-Cas probability of spacer acquisition on the mean diversity of newly generated spacers at the beginning (when S goes extinct, panel A) or at the end (panel B) of the outbreak. For simulations resulting in bacterial extinction at the relevant time, we set diversity to 0. We provide the proportion of bacterial extinction at the beginning (panel C) and at the end (panel D) of the simulations.

The black curve represents the initial diversity of spacers (when S goes extinct) where phage cannot evolve ($\mu = 0$) and the purple, green and red curves when phages can evolve ($\mu = 10^{-8}$, $\mu = 3.4 * 10^{-7}$, $\mu = 10^{-6}$ respectively). On Panel B, the grey line represents the initial diversity. Error bars correspond to 95% confidence intervals and are barely visible due to limited variation.

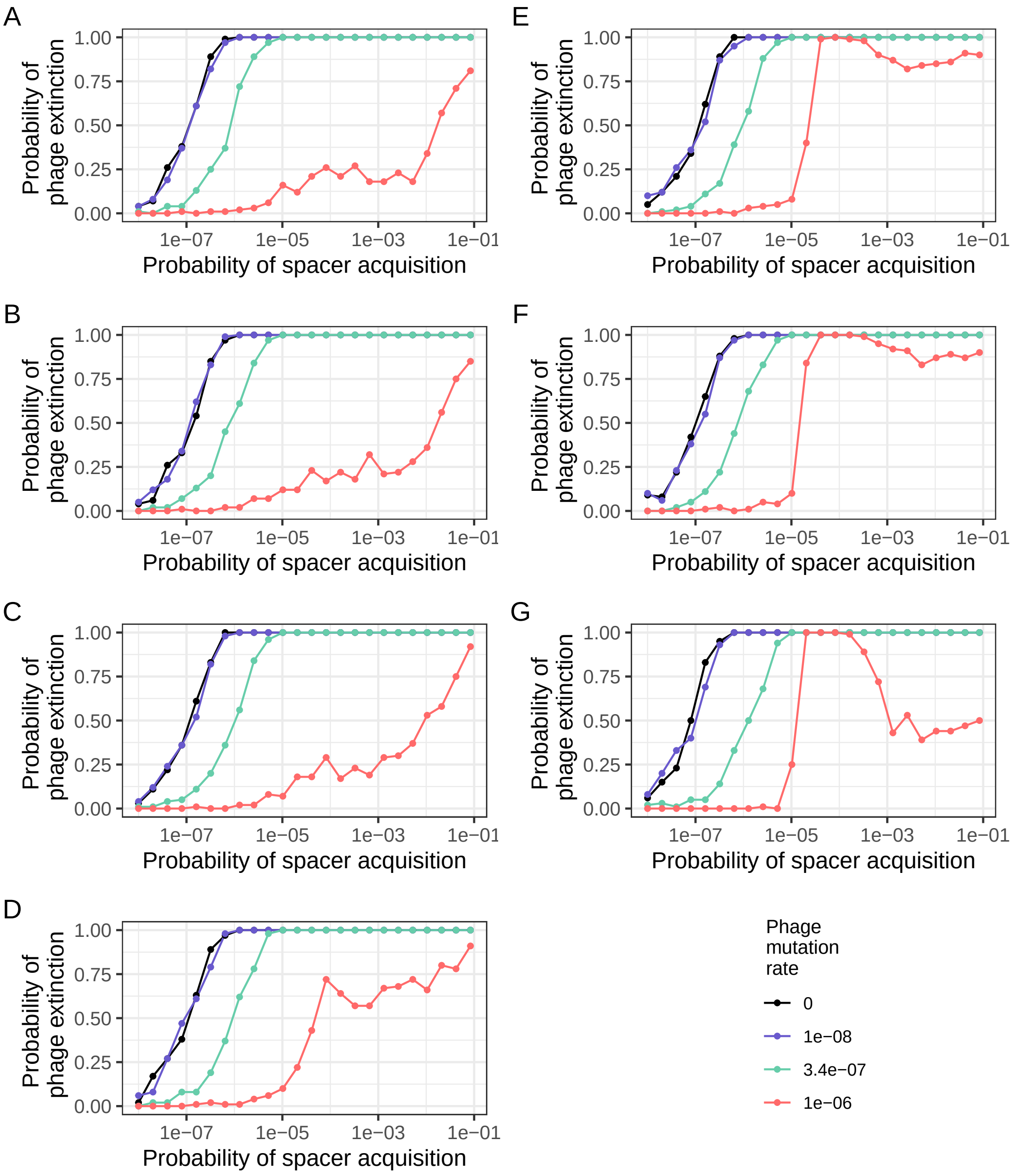

Figure C: Influence of phage infectivity on the epidemiological outcome in the absence of autoimmunity.

Probability of phage survival when infecting bacteria with various probabilities of spacer acquisition. The different colors correspond to different levels of phage evolution (μ): in black, no evolution type III CRISPR-Cas system ($\mu = 0$), in purple $\mu = 10^{-8}$; in green type I/II CRISPR-Cas system $\mu = 3.4 * 10^{-7}$ and in red $\mu = 10^{-6}$. The different panels represent various phage infectivity: A) $\beta = 10^{-2}$, B) $\beta = 10^{-3}$, C) $\beta = 10^{-4}$, D) $\beta = 10^{-5}$, E) $\beta = 10^{-6}$, F) $\beta = 10^{-7}$, G) $\beta = 10^{-8}$

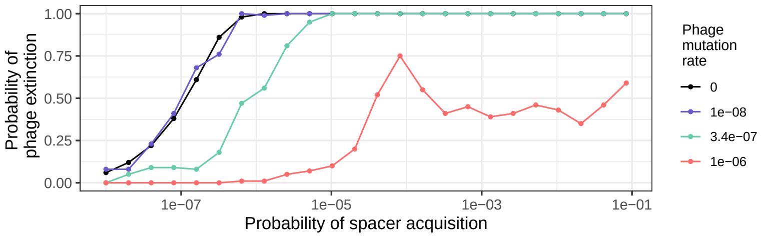

Figure D: Probability of survival, for a phage infecting bacteria with various probabilities of spacer acquisition when phage mutation results in a progeny exclusively composed of escape mutants.

The different colours corresponds to different levels of phage evolution (μ): black, type III CRISPR-Cas system ($\mu = 0$), purple $\mu = 10^{-8}$; type I/II CRISPR-Cas system ($\mu = 3.4*10^{-7}$) and red $\mu = 10^{-6}$.

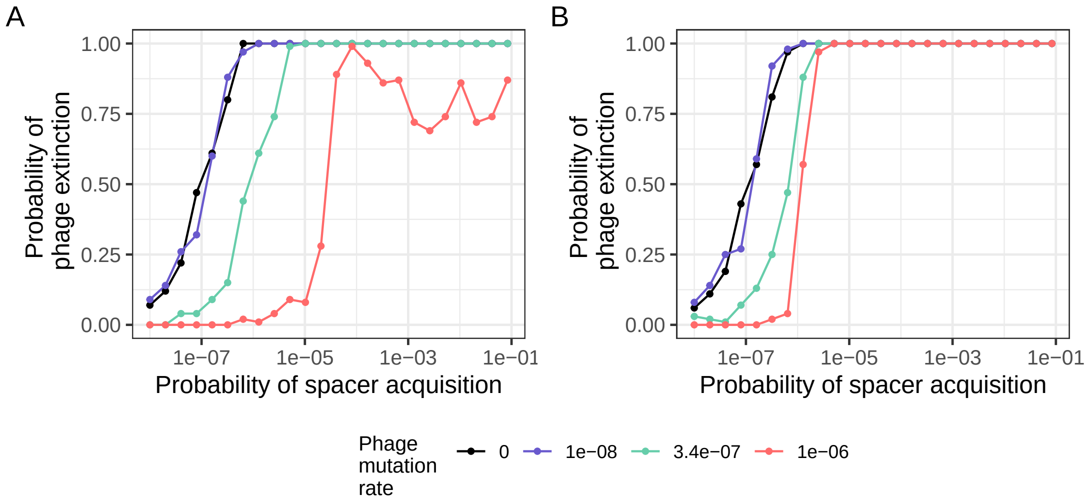

Figure E: Influence of the cost of escaping CRISPR-Cas on the probability of phage extinction in the absence of autoimmunity.

Probability of phage survival when infecting bacteria with various probabilities of spacer acquisition. The different colours corresponds to different levels of phage evolution (μ): black, type III CRISPR-Cas system ($\mu = 0$), purple $\mu = 10^{-8}$; green type I/II CRISPR-Cas system ($\mu = 3.4 * 10^{-7}$) and red $\mu = 10^{-6}$. A) No cost, B) High fitness cost (burst size of mutants equals to 10% of phage WT burst size.)

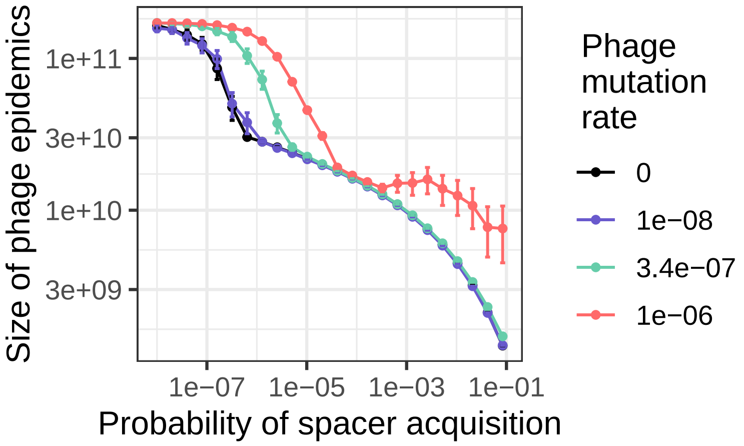

Figure F: Influence of CRISPR-Cas probability of spacer acquisition on the size of a phage outbreak infecting bacteria using CRISPR-Cas immunity.

The colors represent phage evolution: black, type III CRISPR-Cas system ($\mu = 0$); purple, green and red $\mu = 10^{-8}$, type I/II CRISPR-Cas $\mu = 3.4 * 10^{-7}$, $\mu = 10^{-6}$ respectively. Error bars corresponds to 95% confidence intervals.

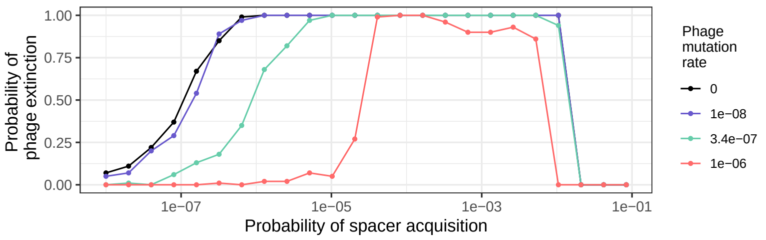

Figure G: Influence of CRISPR-Cas probability of spacer acquisition on the probability of phage extinction in the presence of autoimmunity.

The different colors corresponds to different levels of phage evolution (μ): in black, no evolution, type III CRISPR-Cas system ($\mu = 0$), in purple $\mu = 10^{-8}$; in green, type I/II CRISPR-Cas system $\mu = 3.4 * 10^{-7}$ and in red $\mu = 10^{-6}$.

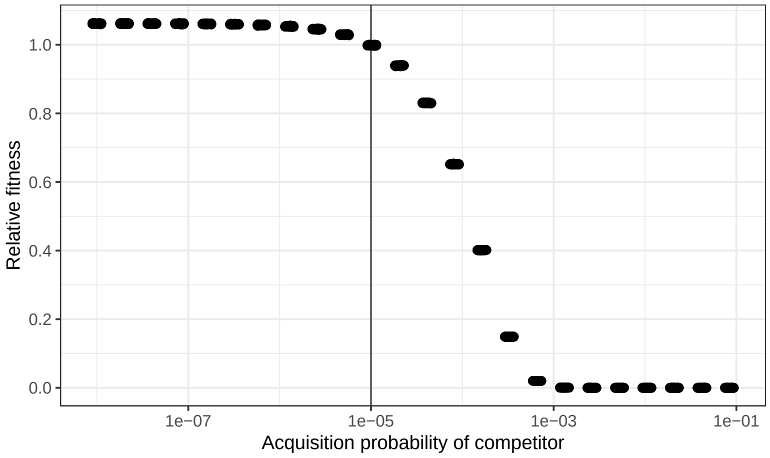

Figure H: Fitness of a bacteria with various probability of spacer acquisition competing against a strain with a probability of spacer acquisition $\alpha = 10^{-5}$ in the absence of phages.

Each competition has been simulated 100 times and for each of them, the relative fitness has been plotted.

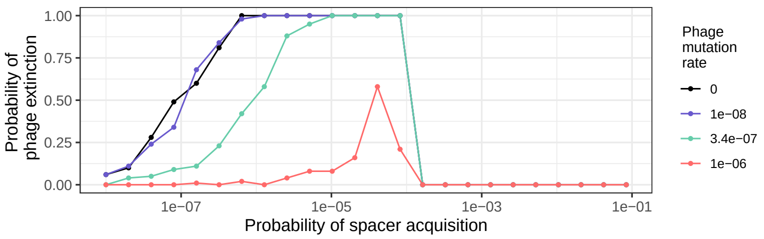

Figure I: Influence of CRISPR-Cas probability of spacer acquisition on the probability of phage extinction with high propensity for autoimmunity (4000).

Probability of phage survival when infecting bacteria with various probabilities of spacer acquisition. The different colors corresponds to different levels of phage evolution (μ): in black, type III CRISPR-Cas system ($\mu = 0$), in purple $\mu = 10^{-8}$; in green type I/II CRISPR-Cas system $\mu = 3.4 * 10^{-7}$ and in red $\mu = 10^{-6}$.
